# Combining Differential Kinematics and Optical Flow for Automatic Labeling of Continuum Robots in Minimally Invasive Surgery

**DOI:** 10.3389/frobt.2019.00086

**Published:** 2019-09-06

**Authors:** Benoît Rosa, Valentin Bordoux, Florent Nageotte

**Affiliations:** ICube, CNRS, University of Strasbourg, INSA, Strasbourg, France

**Keywords:** minimally invasive surgery, continuum robots, computer vision, optical flow, automatic labeling

## Abstract

The segmentation of continuum robots in medical images can be of interest for analyzing surgical procedures or for controlling them. However, the automatic segmentation of continuous and flexible shapes is not an easy task. On one hand conventional approaches are not adapted to the specificities of these instruments, such as imprecise kinematic models, and on the other hand techniques based on deep-learning showed interesting capabilities but need many manually labeled images. In this article we propose a novel approach for segmenting continuum robots on endoscopic images, which requires no prior on the instrument visual appearance and no manual annotation of images. The method relies on the use of the combination of kinematic models and differential kinematic models of the robot and the analysis of optical flow in the images. A cost function aggregating information from the acquired image, from optical flow and from robot encoders is optimized using particle swarm optimization and provides estimated parameters of the pose of the continuum instrument and a mask defining the instrument in the image. In addition a temporal consistency is assessed in order to improve stochastic optimization and reject outliers. The proposed approach has been tested for the robotic instruments of a flexible endoscopy platform both for benchtop acquisitions and an *in vivo* video. The results show the ability of the technique to correctly segment the instruments without a prior, and in challenging conditions. The obtained segmentation can be used for several applications, for instance for providing automatic labels for machine learning techniques.

## 1. Introduction

Continuum robots, contrarily to industrial robots, do not present a succession of joints and rigid links. Instead, a continuously curving, flexible structure is used, in conjunction with actuators that govern its shape. Most of them are tubular in shape, which presents significant advantages for minimally invasive surgery (Burgner-Kahrs et al., 2015). Examples of applications include surgical specialties as wide as endovascular and cardiac surgery (Vasilyev et al., 2015; Devreker et al., 2016), gastroenterology (Berthet-Rayne et al., 2018; Garbin et al., 2018; Zorn et al., 2018), neurosurgery (Swaney et al., 2015), or fetal surgery (Dwyer et al., 2017).

Continuum robots are difficult to model mainly because they use miniature embedded actuation systems. This creates slack, friction, or various nonlinear phenomena which limit the accuracy of existing models (Burgner-Kahrs et al., 2015). Moreover, interactions with the environment impose external forces on the continuum robot. State of the art mechanical models allow computing the robot shape under external loads. Such approaches, however, require integrating sensors in the body of the robot in order to estimate either positions/orientations or interaction forces with the environment (Kim et al., 2014; Mahoney et al., 2016; Shi et al., 2017). Depending on the kind of actuation and size constraints, this is not always possible, and generally not an easy task. Moreover, integrating new sensors in a robot is costly, and requires redesigning existing and available systems.

Surgical vision techniques have been developed as a solution for tracking surgical/continuum instruments and estimating their shape. Vision is especially appealing for endoscopic settings, because a video camera is usually included in the surgical setup, which provides visual guidance to the surgeon. As a result, many works have attempted to segment robotic instruments in endoscopic images. Marker-based solutions have shown promising results for both tool segmentation and 3D pose estimation from 2D images (Cabras et al., 2017). Those approaches, however, modify the instrument body to integrate some markers, thus requiring new developments (such as choice of materials, analysis of cleaning process,…) for *in vivo* use. In order to detect instruments in endoscopic images using marker-less techniques, several approaches make use of color information from the instrument (Doignon et al., 2005), or restrict the search space using constraints related to the medical setup, such as a rigid instrument passing through a trocar in laparoscopy (Voros et al., 2007). Those approaches are however not directly applicable to continuum robots, and/or require human intervention for an initialization step (Pezzementi et al., 2009). In parallel, marker-less approaches using machine learning for pixel-wise instrument segmentation have been developed (Bouget et al., 2017; Bodenstedt et al., 2018). Typically, a training set composed of endoscopic images is manually labeled by an expert, an algorithm learns the links between the labels and some visual features and generalizes them to other surgeries. Such approaches have shown promising results for segmentation as well as for 3D pose estimation (Allan et al., 2018).

**Figure 1:**
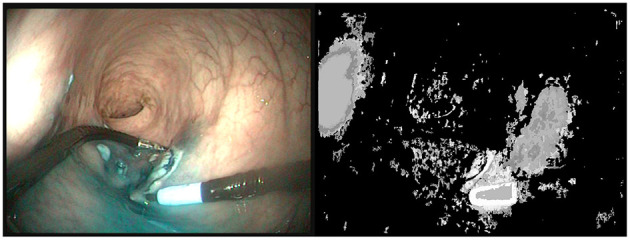
**(Left)** Last image of a 50 images sequence (5s) where the right instrument is moving. **(Right)** Segmentation provided by VideoPCA (Stretcu and Leordeanu, 2015). The salient part of the instrument near the tip is well extracted but a large part of the background is also considered as foreground. Moreover, despite the motion of the instrument, the shaft of the instrument is not considered as foreground.

With the recent uptake of Deep Neural Networks in the Surgical Vision community, interest has grown toward reduced data approaches, i.e., approaches that require less data for training. Transfer learning has been shown to be effective to fine-tune a network using a reduced set of surgery-specific images (Garćıa-Peraza-Herrera et al., 2016). Other approaches using weakly supervised learning (Vardazaryan et al., 2018) or partly unlabeled datasets (Ross et al., 2018) have shown promising results, but some level of manual annotation made by an expert is still required. In order to use fully unsupervised learning, automatic labeling approaches have been developed. These methods are usually based on object saliency detection (Yang et al., 2013; Cheng et al., 2015; Cho et al., 2015). Usually, assumptions are made about what makes an object appear salient, e.g., local contrast differences or a prominent position in the image. Other methods assume that the foreground objects have more complex motions that the background (Stretcu and Leordeanu, 2015). This approach, which extracts information from motion by performing a global principal component analysis of the video sequence is partly related to our work, in the sense that motion is a key element. Nevertheless, as can be seen on Figure 1, the application of the techniques proposed in Stretcu and Leordeanu (2015) does not provide satisfactory segmentations even when tuned to the problem at hand. The probable reason is that in *in vivo* gastroenterology videos the background exhibits complex motions due to physiological motion and interaction of the instruments with the tissues. Therefore, specific tools are needed for handling these videos.

**Figure 2:**
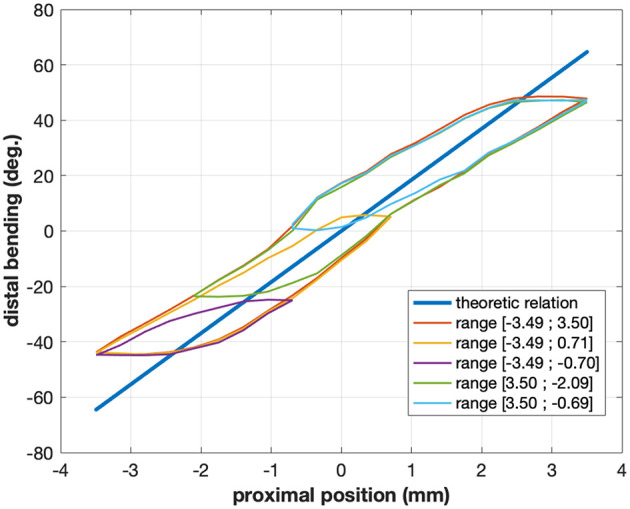
Typical relations between proximal motor position and distal angle for the cable-actuated bending DOF of the STRAS robot (Zorn et al., 2018). The straight line shows the theoretic model relation, while the hysteretical curves show the actual relation for a given configuration of the passive shaft of the robot. Different colors correspond to different ranges of motor motions and highlight the complex behavior of this joint. The actual relation also changes when the shape of the shaft is modified.

Instead of focusing on a pure vision-based approach, this paper makes use of an information which is rarely used in surgical vision: the robot kinematics. As explained before, mechanical models for continuum robots are inaccurate and error-prone. They can nevertheless be combined with image data for enhancing the quality of pose estimation (Tran et al., 2017; Vandini et al., 2017) or tracking (Pezzementi et al., 2009; Reiter et al., 2014). Similarly to approaches purely based on images, current methods making use of kinematic data typically build over image segmentation techniques, using either machine learning methods (with hand-labeled images) or manual initialization. In this paper, we propose a novel method, which makes use of robot forward and differential kinematics, together with optical flow methods, in order to produce pixel-wise image labels. The method is fully automatic, and does not require any human intervention for labeling the data. One key element of using differential kinematics is that in specific cases it is less affected by nonlinearities than the forward kinematic model. Let us consider, for instance, a continuum robot with a single bending section actuated with cables. One of the main contributors to nonlinearities is friction (Do et al., 2015; Ha et al., 2018), which is most important when the actuator changes direction (see Figure 2). At those moments, the speed computed by the model may be non-zero, while the actual robot speed will be zero due to friction. The difference between the model-predicted and actual robot speed will be integrated over time, leading to large position errors in the kinematic model. Outside of those moments, however, the differential kinematic model will be correct, while the forward kinematics may keep a large error due to previously integrated errors. This behavior is illustrated in Figure 2 by the case of the robot considered for experimental validation in this study. The curves for the model-predicted (straight line) and actual link between the position of the motor driving the instrument bending and the distal bending angle do not superimpose well, with large errors of up to 20°, whereas the slopes of the curves, which are linked to the differential kinematics, are quite similar as long as the considered configuration is far away from a change of direction for the motor (low slope areas inside the hysteresis). This effect is more complex when considering multi-DOF continuum robots, but the validity of the differential kinematic model can nevertheless be considered better far away from direction changes in the motor input.

The paper is organized as follows: first, section 2 describes the technical foundations of the method. Then, the proposed method is described in depth in section 3. Sections 4, 5, respectively, present the experimental validation conditions and the results, and section 6 concludes the paper.

## 2. Differential Kinematics and Optical Flow

This section presents the theoretical foundations needed for presenting the method in the next section. General results concerning forward and differential kinematics of continuum robots are presented, as well as how they can be used for generating virtual optical flow images.

### 2.1. Continuum Robot Kinematics

Let us consider the model of a continuum inextensible robot as a function *g*(*q, s*) ∈ *SE*(3), which is a homogeneous transformation function describing the position and orientation of the robot central line for given joint variables *q* ∈ ℝ^*n*^ and an arc-length *s* ∈ [0, *L*], L being the total length of the robot. *x*(*s*) ∈ ℝ^3^ is the translational part of *g*, i.e., the position of the robot centerline at an arc-length *s*. *g* and *x* are defined in the robot base frame *r*.

Various methods are available in the literature to compute the robot forward kinematics, either by direct computation if constant curvature can be assumed (Webster and Jones, 2010), or using iterative schemes and Cosserat rod theory for more complex cases (Dupont et al., 2010; Burgner-Kahrs et al., 2015). Once the forward kinematics has been obtained, the differential kinematics represented by the Jacobian *J*(*q, s*) can be obtained either analytically or by using the finite differences method :

(1) $$\begin{array}{l} J\left(q,s\right)=\left[\begin{array}{c} \frac{\partial g\left(q,s\right)}{\partial q_{i}}\\ \vdots \\ \frac{\partial g\left(q,s\right)}{\partial q_{n}} \end{array}\right] \end{array}$$

Like the forward kinematics, the Jacobian matrix depends both on the current joint configuration *q* and on the considered arc-length *s* of the robot. For a known (small) time step, one can compute the robot displacement ẋ for any point *s* along the robot centerline by using $ẋ\left(s\right)=J\left(q,s\right)\overset{\cdot }{q}$.

**Figure 3:**
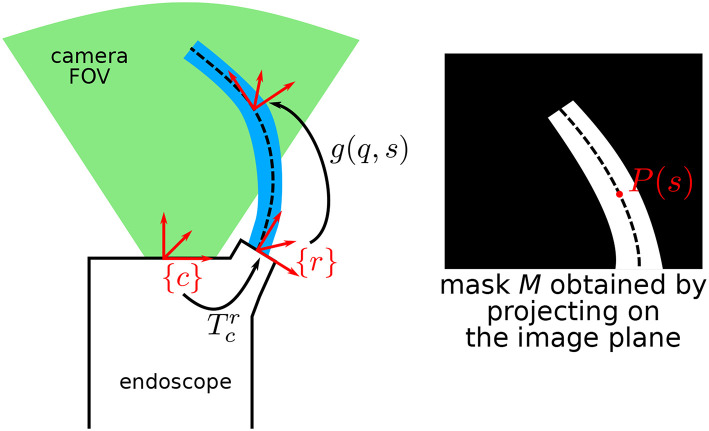
Schematic describing the general situation, where a continuum robot is in the field of view (FOV) of an endoscopic camera. *P*(*s*) is the projection of the centerline of the robot at arc-length *s* onto the image plane.

In this paper, without loss of generality, we consider the case depicted in Figure 3, where a cable-actuated robot is inserted in an endoscope (Zorn et al., 2018). In this case, the joint variables *q* are the insertion length of the robot in the channel, the differential length of the two antagonist cables used for bending the robot, and the rotation of the robot base around the axis of the channel. Using the formalism presented in Webster and Jones (2010), together with robot specific properties (i.e., diameter and length of the actuated and passive sections), one can compute the robot forward and differential kinematics analytically for any joint position *q*. Details about the kinematic model equations of the considered robot are available in Appendix.

Let us further introduce the vector δ ∈ ℝ^*n*^, which can be added to *q* in order to change the robot pose. δ will be used in the following of this paper as a corrective vector on *q*, which will be optimized in order to extract relevant pixel-wise labels of the tool. As such, the positions *q* and *dq* (or $\overset{\cdot }{q}$) always correspond to the nominal values.

Finally, let us note that in the following we are only interested about 3D positions and linear velocity. The Jacobian matrix *J* is therefore reduced to a 3 × 3 matrix, expressed in the robot base frame.

### 2.2. Optical Flow

Optical flow refers to a set of computer vision methods, which have been developed to infer the displacement between two images. Classical methods are typically keypoint-based, thus only providing the flow in regions of the image with sufficient texture. Densification methods based on various criteria have been proposed in the literature to solve this problem (Wedel and Cremers, 2011). Recently, deep learning approaches have shown promising results for fast and accurate dense optical flow estimation (Ilg et al., 2017). Interestingly, such approaches can learn from 3D rendered scenes, for which an accurate ground truth optical flow is known, and generalize well to other types of video images.

**Figure 4:**
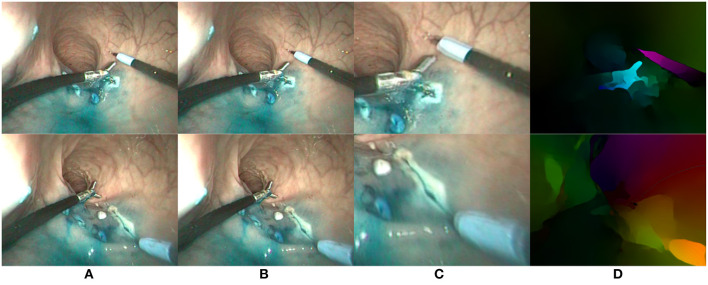
Two examples (one per row) of optical flow estimation on *in vivo* endoscopic images. **(A)** First image; **(B)** Second image; **(C)** Zoom on differential image; **(D)** FlowNet2 output flow map.

In this paper, we use the state of the art FlowNet2.0 algorithm (Ilg et al., 2017), which can compute dense flow maps in a few hundred milliseconds given a pair of input images. An example with *in vivo* endoscopic images is shown on Figure 4. For all flow values considered later on, we use the magnitude-direction model, where the magnitude represents the norm of the flow vector while the direction represents the angle of the flow vector w.r.t. a horizontal reference vector. For visual display of the optical flows, we use the HSV color space, where the direction is mapped to the hue H, the magnitude to the value V, and the saturation is set to the maximum for enhanced visualization. In the following of the paper, optical flow images obtained using the FlowNet2.0 algorithm are noted $\hat{F}$.

### 2.3. Virtual Optical Flow Rendering

In order to put the robot differential kinematics in relation with the optical flow $\hat{F}$, we introduce the notion of virtual optical flow maps. Let us consider a time instance *t*, at which the robot joint values *q*(*t*) are known. As described above, one can compute the forward kinematics of the robot *g*(*q*(*t*), *s*), as well as the Jacobian matrix at any point along the robot shaft, *J*(*q*(*t*), *s*) (in the following the dependency to *t* is omitted for clarity). Moreover, let us consider that the endoscopic camera is calibrated with intrinsic parameters *K*, and that radial distortions are compensated. The hand-eye calibration from the camera base frame to the robot base frame, given by the homogeneous matrix $T_{c}^{r}$ or equivalently rotation $R_{c}^{r}$ and translation $t_{c}^{r}$, is considered as known.

From the above-described situation, one can first project the robot in the estimated pose onto the image. For any point of arc-length *s* ∈ [0, *L*], one can obtain the projected position on the image plane *P*(*s*) by computing:

(2) $$\begin{array}{l} P_{c}\left(s\right)=\left(\begin{array}{c} X\left(s\right)\\ Y\left(s\right)\\ Z\left(s\right) \end{array}\right)=R_{c}^{r}\;g\left(q,s\right)+t_{c}^{r} \end{array}$$

(3) $$\begin{array}{l} P_{hom}\left(s\right)=K\left(\begin{array}{c} \frac{X\left(s\right)}{Z\left(s\right)}\\ \frac{Y\left(s\right)}{Z\left(s\right)}\\ 1 \end{array}\right) \end{array}$$

*P*_*c*_(*s*) represents the 3D position of the robot centerline at arc-length *s* expressed in the camera frame, and *P*_*hom*_(*s*) is the projection in the image in homogeneous coordinates. Coordinates of *P*(*s*) in pixels in the image are the first two rows of *P*_*hom*_(*s*). By considering that the diameters of the different parts of the robot are known by design, the whole robot shape can be rendered on the image plane, obtaining a binary image mask M, as shown on Figure 3.

One can use a similar principle for generating virtual optical flow images. The overall idea is to populate the mask M with the projections of the local 3D speed values onto the image plane. This is done by discretizing the central-line and, for each of the obtained discrete values, by assigning the projection of the centerline speed *v*(*s*) to the local area of the mask around point *P*(*s*). *v*(*s*) is obtained as:

(4) $$\begin{array}{l} v\left(s\right)=J_{im}\left(P_{c}\left(s\right)\right)R_{c}^{r}\;J\left(q,s\right)dq \end{array}$$

(5) $$\begin{array}{l} \text{with }J_{im}\left(P_{c}\left(s\right)\right)=K_{1:2,1:2}\left(\begin{array}{ccc} \frac{1}{Z\left(s\right)} & 0 & -\frac{X\left(s\right)}{Z{\left(s\right)}^{2}}\\ 0 & \frac{1}{Z\left(s\right)} & -\frac{Y\left(s\right)}{Z{\left(s\right)}^{2}} \end{array}\right) \end{array}$$

*J*_*im*_(*P*_*c*_(*s*)) is the so-called image Jacobian, which relates the 3D velocity of a physical point to the apparent 2D velocity of its projection in the image plane. This computation assumes that time-steps are small enough for the Jacobian-based computation to be valid. The resulting image is called F^*v*^.

A few properties of the virtual optical flow map are worth noting. First, the flow magnitude is set to 0 outside of M. This is because the robot kinematics does not provide any information allowing us to infer the speed values for the environment (the organs and tissues).

**Figure 5:**
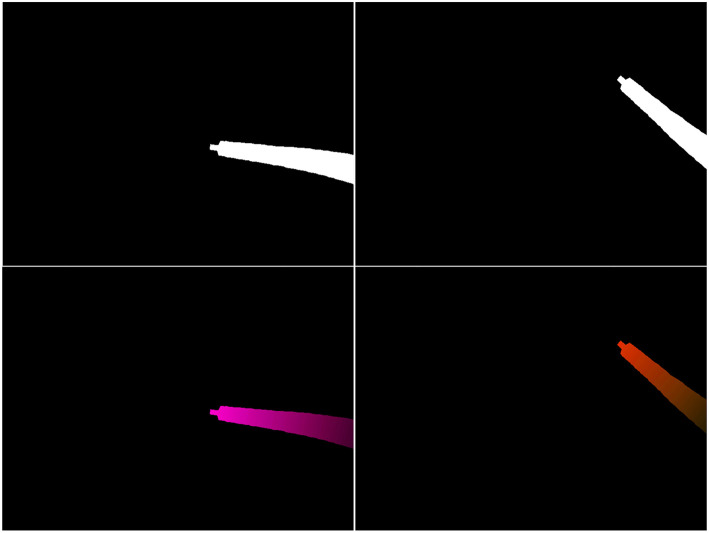
Example of projected mask M (top row) and virtual optical flow maps F^*v*^ (bottom row). **(Left)** Output of the kinematic model for a given *q* and *dq*. **(Right)** Output of the model for *q* + δ and the same *dq*.

Second, the obtained virtual flow map will depend on both the robot pose *g*(*q, s*) and on *dq*. Joint values *q* will typically be obtained from sensors placed on the different motors driving the robot joints. *dq* can then be obtained by differentiation of *q* at time *t*, such as *dq*(*t*) = *q*(*t* + *dt*) − *q*(*t*). If we now apply a corrective vector δ on *q* (see definition of δ in section 2.1), the robot shape equation will then be described by *g*(*q* + δ, *s*), affecting the pose of the robot and therefore the mask M (top-right on Figure 5). Moreover, the robot Jacobian will also be affected, which means the flow *values* in *F*^*v*^ will also change, as illustrated on Figure 5 (bottom row).

## 3. Optimization-Based Automatic Labeling

This section presents the optimization-based approach which was developed for automatic labeling of continuum robots in endoscopic images. The proposed method uses the tools described in section 2 in an iterative optimization scheme, in order to infer the mask M that best fits with the continuum robot in the images.

### 3.1. Optimization

**Figure 6:**
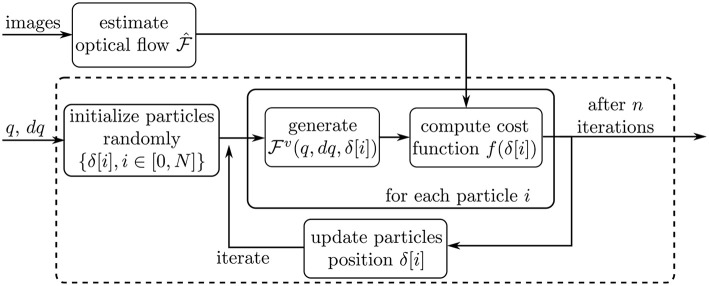
Schematic describing the optimization workflow. The dashed box outlines the Particle Swarm Optimization process.

This subsection describes the actual optimization routine applied on each processed image. The overall workflow of the optimization is depicted on Figure 6. First, the dense optical flow $\hat{F}$ is computed by using two consecutive images in the video sequence. Second, the nominal robot pose parameters *q* and the value of *dq* are extracted from sensors at the motors side, as described above. Finally, the iterative optimization process is started. The optimizer iterates on the variable δ defined in the previous subsection, in order to minimize a cost function *f*. The cost function and optimization algorithm are described in the following subsections.

#### 3.1.1. Cost Function

We define the cost function as a sum of four terms (all terms depend on δ but the dependence is omitted in the following for clarity):

(6) $$\begin{array}{l} f=\alpha f_{direct}+\beta f_{h1}+\gamma f_{h2}+f_{q} \end{array}$$

*f*_*direct*_ is a direct comparison of optical flow values between the estimated and virtual optical flow. It is defined as the normalized average of the direction and magnitude differences between $\hat{F}$ and F^*v*^:

(7) $$\begin{array}{l} f_{direct}=\frac{1}{2}\overset{1}{\underset{i=0}{\sum }}\frac{1}{C_{i}}\frac{\sum_{x,y}|\hat{F}\left(x,y,i\right)-F^{v}\left(x,y,i\right)|M\left(x,y\right)}{\sum_{x,y}M\left(x,y\right)} \end{array}$$

where *x, y* are pixel indices, and *i* represents the third dimension of the optical flow maps, 0 for direction and 1 for magnitude values [e.g., F^*v*^(*x, y*, 0) is the virtual optical flow direction at pixels x,y]. *C*_*i*_ are normalization terms set at the maximum possible values of the directions differences (180°, which is the maximal angular difference due to circular wrapping) and magnitude, respectively. This ensures that *f*_*direct*_ values are always between 0 and 1.

*f*_*h*1_ compares histograms of flow between the inside and outside of the current robot mask. The intuition behind this term of the cost function is that the robotic arm should move independently from the environment (which, in surgical situations, moves following complex physiological motion patterns). Therefore, histograms of flow values should be uncorrelated if the projected mask M is well-aligned with the robot in the image. On the contrary, if the mask partially overlaps with the environment, flow values inside and outside the mask will both contain values of speeds from the environment and from the robot, and will thus be correlated. In order to express this as a cost function, let us consider the mask M obtained by projection of the robot shape on the image. The mask can be enlarged by performing a morphological opening operation with a structuring element *e*. We define N = M⊕ *e* − M, which covers an area around the original mask M (i.e., a contour). We then compute the histogram of flow values in the regions defined by M and N. For a given flow image F, the histogram *p*_F_ is obtained in three steps. First, the directional part of F is quantized into *N* bins, yielding an array *B* with the bin values and a 2D array F_*d*_ containing the quantized orientation values. The histogram can then be computed in two steps :

(8) $$\begin{array}{l} m_{F}\left(i\right)=\underset{x,y}{\sum }\delta_{B\left(i\right)}^{F_{d}\left(x,y\right)}F\left(x,y,1\right) \end{array}$$

(9) $$\begin{array}{l} p_{F}\left(i\right)=\frac{m_{F}\left(i\right)}{\sum_{x,y}m_{F}\left(i\right)} \end{array}$$

where *i* is the bin number, and δ is the Kronecker symbol. The histograms computed this way are normalized flow direction histograms weighted by the flow magnitude (Equation 8). The weighting gives more importance to local flow directions which are associated with a large magnitude. This allows to attenuate the impact of directions which are ill-defined when the magnitude approaches zero. The histograms are then subsequently normalized (Equation 9). In order to compute *f*_*h*1_ we calculate the flow histograms inside and outside the robot mask, i.e., $p_{\hat{F}⊙M}$ and $p_{\hat{F}⊙N}$, where ⊙ is an element-wise multiplication operator. Since the histograms are normalized, they can be compared using standard probability density comparison metrics. In this work, we chose the Jensen-Shannon distance. The Jensen-Shannon distance is defined as the square root of the Jensen-Shannon divergence, which is itself defined as the symmetric version of the Kullback-Leibler (KL) divergence (Cha, 2007) :

(10) $$\begin{array}{l} \text{JS}\left(a,b\right)=\sqrt{\frac{KL\left(a,\frac{a+b}{2}\right)+KL\left(b,\frac{a+b}{2}\right)}{2}}. \end{array}$$

Compared to the KL divergence, it is symmetric, bounded between 0 and 1, and satisfies the triangle inequality. In our case, since we aim at minimizing the correlation between histograms of flows inside and outside the robot mask, the cost function *f*_*h*1_ is defined as:

(11) $$\begin{array}{l} f_{h1}=1-\text{JS}\left(p_{\hat{F}⊙M},p_{\hat{F}⊙N}\right). \end{array}$$

*f*_*h*2_ is similar to *f*_*h*1_ in the sense that it also compares histograms between the masks M and N. It does, however, directly compare color histograms from the source image. The underlying assumption is that the background is more or less visually uniform, and likely visually different from the robot appearance. Therefore, for computing *f*_*h*2_ we compare inside and outside histograms using simple color features. Taking the input image I and the mask M as input, we start by representing the image in the HSL color space. HSL is similar to the well-know HSV space, except that Luminance replaces the Value. Luminance is a weighted sum of the R, G, and B components using factors representative of the human perception, so that *L* = 0.2125*R* + 0.7154*G* + 0.0721*B*, and is therefore closer to human light intensity perception than the simple Value (Plataniotis and Venetsanopoulos, 2000). The Jensen-Shannon distance is computed for each of the components of the HSL representation using the same process as for *f*_*h*1_. We define *f*_*X*_ = 1 − JS(*p*_*X*⊙M_, *p*_*X*⊙N_), where *X* stands for H,S, or L. *f*_*h*2_ is then expressed as:

(12) $$\begin{array}{l} f_{h2}=\sqrt[{3}]{f_{H}f_{S}f_{L}} \end{array}$$

Finally, the last term of the cost function, *f*_*q*_, is a penalization term on the pose parameters themselves. It is defined as :

(13) $$\begin{array}{l} f_{q}=\lambda \overset{dim\left(\delta \right)}{\underset{i=0}{\sum }}\frac{1}{1+exp\left(a\left(\delta_{i}+b_{i}\right)\right)}\frac{1}{1+exp\left(a\left(-\delta_{i}+b_{i}\right)\right)} \end{array}$$

where λ, *a*_*i*_ and *b*_*i*_ are constants. This function allows penalizing large deviations δ. If *a* is chosen large enough, the function is almost flat and with very low values when all components δ_*i*_ ∈ [−*b*_*i*_, *b*_*i*_], while quickly (and smoothly) reaching value λ as one component of δ approaches one of the bounds. Specific values of *a* and *b*_*i*_ must be chosen depending on the allowed pose parameters variations, which can themselves be linked to the estimated errors bounds in the mechanical model. Any λ ≫ 1 is acceptable, since *f*_*direct*_, *f*_*h*1_ and *f*_*h*2_ are all bounded by [0, 1].

#### 3.1.2. Optimization Algorithm

The above-defined cost-function is a sum of four terms, which involve local computations (which is mainly due to the fact that F^*v*^ only contains informations inside the projected mask M). Therefore, it cannot be assumed to be either smooth or convex. For this reason, we chose a global stochastic optimization algorithm, Particle Swarm Optimization (PSO) (Poli et al., 2007). PSO generates a number *N* of candidate solutions by random sampling of the search space, and makes those so-called particles evolve over time, based on simple formulae governing their position and velocity. The algorithm makes little assumptions about the problem to be optimized, and does not require computing the gradient of the cost-function, which makes it well-suited for global optimization of potentially non-smooth functions. Moreover, within an iteration, cost function evaluations for each particle are totally independent, which makes the algorithm parallelizable (Mussi et al., 2011). Finally, PSO can return the overall minimum as well as the values and cost function evaluations for each independent particle. As we will see in the next subsection, this property is interesting in our context.

### 3.2. After Optimization: Enforcing Local Consistency

Since the PSO algorithm is stochastic, it does not guarantee convergence to the true minimum. Moreover, the cost function is based on some assumptions which may be violated. This is especially the case for the hypothesis on the independence of optical flows between the instrument and the environment, which underlies the histogram comparison term *f*_*h*1_. For instance, the optical flow may be misestimated, or parts of the background may temporarily move in synchrony with the robotic instrument. In these cases, the optimization minimum may not correspond to the actual pose parameters of the instrument.

In order to detect and filter out those false convergences, we introduce some metrics meant to assess the consistency between successive optimization results. Let us note *R*(*t*) = {(δ_*j*_(*t*), *f*(δ_*j*_(*t*)), *j* ∈ [1, *N*]} the output of the PSO algorithm after convergence, at time instance *t*. This set represents the positions of the *N* particles after evolving through the PSO algorithm, with their associated cost function values. The overall minimum is *f*_*min*_(*t*) = argmin_*j*_*f*(δ_*j*_(*t*)).

In order to enforce local temporal consistency, we compare sets of results at successive time instances. Let us note those time instances *t*_1_ and *t*_2_. After independent optimization using the above-described algorithm, the respective result sets are *R*(*t*_1_) and *R*(*t*_2_).

Let us note *T* = *g*(*q*(*t*), *L*) and *T*_*o*_ = *g*(*q*(*t*) + δ, *L*) with *t* ∈ {*t*_1_, *t*_2_}. *T* and *T*_*o*_ are homogeneous transformations from the robot base to the tip before and after correction with a vector δ, respectively. The relative tip pose correction can then be computed as $dT=T^{-1}T_{o}$. The translational part of *dT* is noted *dV*. This vector represents the tip displacement created by applying the correction δ, and expressed in the initial tip frame of the robot. It is dependent on both the considered frame (time *t*), as well as on the value of δ. Its full notation is thus *dV*(*t*, δ)

Finally, we define the tip local distance *D*(*i, j*) as :

(14) $$\begin{array}{l} D\left(i,j\right)=||dV\left(t_{1},\delta_{i}\right)-dV\left(t_{2},\delta_{j}\right)|| \end{array}$$

where *i* and *j* are indices of elements in *R*(*t*_1_) and *R*(*t*_2_). Large values of *D*(*i, j*) indicate an important change of estimated offsets of the pose parameters between images at *t*_1_ and *t*_2_. The distance *D*(*i, j*) is better suited for estimating closeness of results than directly comparing values of δ because it is in the task space, therefore less sensitive to kinematic singularities which may arise (for instance, if the robotic arm is straight, any rotation of the arm leads to the same pose, and the PSO may output many particles with low cost values but with very different rotation values).

The algorithm for local temporal consistency check can be described the following way:

- Initialization: sort the elements of *R*(*t*_1_) and *R*(*t*_2_) according to their cost function values. The elements with lower costs are set as first elements: (δ_1_(*t*_1_), *f*_1_(*t*_1_)) and (δ_1_(*t*_2_), *f*_1_(*t*_2_))
- Compute *D* = *D*(1, 1) and $f_{c\text{\_}ref}=\sqrt{f_{1}\left(t_{1}\right)f_{1}\left(t2\right)}$
- For (*i, j*) in [1, *N*]^2^:
  - Compute *D*(*i, j*) and $f_{c}\left(i,j\right)=\sqrt{f_{i}\left(t_{1}\right)f_{j}\left(t2\right)}$
  - If *D*(*i, j*) < *D* and *f*_*c*_(*i, j*) < 2*f*_*c*_*ref*_ set *D* = *D*(*i, j*)

At the end of the process, the couple of particles from both optimization outputs having the closest *D* distance, as well as a pooled cost function *f*_*c*_ low enough (< 2*f*_*c*_*ref*_) is selected. The selected particle from *R*(*t*_1_) is the result of the optimization after consistency check. The particle from *R*(*t*_2_) is not used as the information from the second image is redundant with the information obtained from the first image. Note that during the iterations *f*_*c*_*ref*_ is never reset. This is done in order to select a *good enough* couple of particles (i.e., with a pooled cost function *f*_*c*_ close to the minimum value), while having consistent δ corrections.

## 4. Experimental Validation

This section presents the experimental validation details, the robotic system used, the implementation details, as well as the various testing conditions.

### 4.1. System and Images

This study makes use of the STRAS robotic arm. STRAS is a flexible endoscopy robot designed for complex endoluminal operations such as Endoscopic Submucosal Dissection (De Donno et al., 2013; Zorn et al., 2018). The robot arms are 3.5 mm diameter flexible cable-actuated instruments. Each arm has 3° of freedom controlled by independent motors: the insertion of the instrument in the channel, the rotation of the instrument along its own shaft, and the bending of the tip. The motors encoders are used to compute the joint variables *q*, which in turn can be used for computing the forward and differential kinematics, as detailed in Appendix.

During the operation of the robot, the surgeon sends commands through a dedicated user interface to teleoperate the robot, while using the images from the endoscopic camera for guidance. This system has a fixed focal, and therefore camera calibration can be performed in the lab and remain valid during *in vivo* use. We used standard calibration procedures from Zhang (2000) for obtaining the camera calibration matrix *K* as well as the distortion parameters (5 parameters distortion model). Images are distortion-corrected in real-time using the OpenCV library, therefore points in the 3D space can be projected on the distortion-corrected image using the calibration matrix *K*.

In order to project 3D points from the robot model onto the images, the hand-eye calibration matrix $T_{c}^{r}$ is also required. In this work, since the flexible arms pass through the working channel of the endoscope, we set $T_{c}^{r}$ to its nominal value using the CAD model of the endoscope. While this is not exact –especially in the case where the robot tip is very close or very far from the exit of the working channel, as shown in Cabras et al. (2017), it was considered sufficient in this study. It would be possible to also optimize parameters of the hand-eye transformation at the cost of adding a few more optimization variables, but this was left out of the scope of the present paper. In fact, the uncertainty of the hand-eye calibration is often partly compensated by the offset δ, leading to a good 2D projection of the tool onto the image plane. Further research will consider optimizing the hand-eye calibration parameters in the future, especially for full 3D pose estimation of the robot.

**Figure 7:**
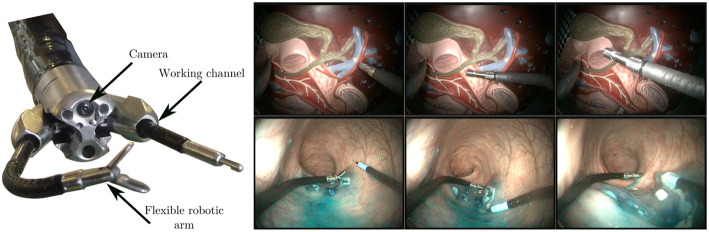
**(Left)** View of the tip of the STRAS robot, showing the endoscopic camera, the deploying working channels, and the flexible robotic arms. **(Right)** Sample images from the lab (top row) and *in vivo* (bottom row) datasets, featuring robot and environment movement, as well as tool/tissue interaction. Note that the visual appearances of the instruments are significantly different between both sets.

To validate the proposed algorithm, we acquired two different datasets (see Figure 7). The first one was generated on the benchtop, by teleoperating the robot in front (and in interaction with) a plastic model of the human digestive anatomy (1,000 images, 100 s long). This model was manually moved by an operator in all directions during the robot teleoperation in order to simulate physiological movements. Displacements obtained in the images are complex because the model features 3D shapes.

The second dataset (2,000 images, 200 s long) was obtained during an *in vivo* experiment in a porcine model. The surgeon performs an actual colorectal Endoscopic Submucosal Dissection by teleoperating the STRAS robot. The study protocol for this experiment was approved by the Institutional Ethical Committee on Animal Experimentation (ICOMETH No.38.2011.01.018). Animals were managed in accordance with French laws for animal use and care as well as with the European Community Council directive no. 2010/63/EU. Images feature smoke, tissue cutting, and specular reflections. The dataset is particularly challenging because images were not acquired at the beginning of the surgery. The instrument is almost always in interaction with the tissues, which increases errors in the kinematic models due to external forces and wrenches imposed on the instrument. Note that this dataset features two robotic arms which were used during the surgery, but only the kinematic information from the right arm was used.

### 4.2. Implementation Details

The proposed algorithm was implemented in Python, using tools from OpenCV (Bradski, 2000) and numpy (Oliphant, 2007) for images and arrays manipulations, respectively. Optical flow was computed using the publicly available Tensorflow GPU code from the authors of the algorithm (Ilg et al., 2017) and the full pre-trained FlowNet2 model. Both video and optical flow images have a resolution of 760 × 570 pixels, and the motors encoders outputs and video images were synchronized with a time step *dt* = 100 ms.

Particle Swarm Optimization was implemented in python using a combination of the pyswarm library (Lee, 2014) and pathOS for multiprocessing support (McKerns et al., 2011). The algorithm was setup using different sizes of the swarm *N* and different iterations numbers *i*. Other parameters of the PSO algorithm, *ϕ*_*p*_ and *ϕ*_*g*_, which are scaling factors determining the evolution of the particles at each iteration (how far away from the particle and swarm's best known position, respectively; Poli et al., 2007) were set to their default value of 0.5. Finally, after the consistency check, the final result obtained was considered of poor quality and therefore discarded if it did not satisfy the following constraints: *f*_1_(*t*_1_) < 0.4 and *f*_1_(*t*_2_) < 0.4 and *D* < 2*mm*. These thresholds have been set to be conservative and reject most incorrect/inconsistent results. Further research is needed to determine optimal values.

For the cost function, α, β, and γ were, respectively, set to 0.1, 0.25, and 0.5. Those values were chosen in an *ad-hoc* fashion based on the relative scale exhibited by the different terms on the considered pilot images, so as to confer to them the same relative importance in the cost function. For computing *h*_1_ and *h*_2_, 10 successive dilations with a 5 pixels wide rectangular structuring element *e* were performed. *f*_*q*_(δ) was computed with λ = 10, *a* = 200, and *b* set in such a way that the search space is bounded by ±35° for the bending angle at the tip of the robot and the rotation angle, and ±5 mm for the robot insertion in the channel. These values were chosen larger than the observed hystereses effects on each joint (see on Figure 2 for bending), in order to take into account potential errors originating from robotic homing positions, hand-eye calibration errors, or from external forces applied on the instrument.

The algorithm was run on a Intel Xeon processor with 20 cores in order to take advantage of the multiprocessing implementation of PSO. FlowNet was run on a Nvidia 1080GT GPU. Average runtime of the full optimization routine for one pair of images was between 9 and 15 s depending on the parameters of the PSO algorithm. This is at least one or two orders of magnitude too slow for online use of the algorithm. However, this is mostly due to the multiple array initialization/manipulation routines used in the cost function evaluation, which is in turn called a large number of times during the optimization process. A careful, optimized C++ implementation, or a parallel implementation using Cuda on a GPU may provide significant speedups.

### 4.3. Metrics and Evaluation

In order to evaluate the output of the algorithm, images were manually annotated in the form of binary masks. In total, 394 images were manually annotated in the benchtop dataset, and 359 in the *in vivo* dataset. Using those binary masks as ground truth, masks M generated by the kinematic model after applying the optimized correction δ were then compared using the Precision, Recall, and Intersection over Union (IoU) metrics.

In order to fully characterize the algorithm using the above-defined metrics, several parameters were explored.

#### 4.3.1. Sampling Strategy

Since the overall optimization runs in 9 to 15 s, it is unlikely that we will be able, even with important code optimization, to make it run in real time. This is, however, not an important problem since successive images are redundant in content and therefore only a few images should be used for generating high quality labels for subsequent classifier training. In other words, we need to sample images from the dataset. We evaluated two sampling strategies. The first one, which we call *S*_*rand*_, is a completely random sampling strategy. The second one, which we call *S*_*sel*_ is based on the joint angles input. Since nonlinearities in the robot kinematics are more likely to appear at direction changes (see Figure 2), we select images which are sufficiently far from those direction changes. Practically, *q* is differentiated in order to obtain $\overset{\cdot }{q}$. To remove local sensor noise, smoothing is performed using the standard 3rd order Savizky-Golay algorithm. A simple detection of sign changes in the last 500 ms is then performed (zero being considered as a sign change). One should note that this method only uses information obtained from *before* the current timepoint, and is thus usable in real-time settings.

Finally, promising images from the previously selected images are kept if:

- The projection of the robot tip at the nominal pose and shape provided by *g*(*q, L*) is not close to the borders of the image, and
- If the robot speed at the nominal tip position, projected in the image plane, is above a certain threshold.

These conditions are used to avoid situations where the robot is actually outside the field of view because of modeling errors, or where the real optical flow is very low due to projection conditions (typically when the velocity of the robot is aligned with the line of view). Practically, we use a window of 3/4 of the image size for the first condition, and 2 pixels of robot optical flow speed magnitude for the second condition.

For fairness of evaluation, the two sampling strategies should present the same number of images. *S*_*sel*_ being the most restrictive, it was applied to the beginning of the video (the first 2 min for the *in vivo* case, the first minute for the benchtop case), thus providing 33 selected images for the *in vivo* dataset and 36 images for the benchtop dataset. The same number of images was then extracted using random sampling on the same part of the videos.

#### 4.3.2. Optimization and Consistency Check

Another parameter which may influence the results is the application or not of the consistency check. In order to test our hypothesis that our optimization algorithm is effective and that our proposed consistency check procedure helps filtering out bad results, we considered three different test cases. *O*_*no*_ represents the results obtained on a given test using the uncorrected kinematic model, i.e., with δ = [0, 0, 0]. *O*_*optim*_ represents the case were the optimization algorithm is run on a single image, without performing the consistency check. Finally, *O*_*optim*+*cc*_ represents the case were the full algorithm is run, performing the optimization on two consecutive images in order to apply our consistency check procedure.

#### 4.3.3. PSO Parameters and Randomness

As explained above, we use the PSO algorithm for exploring the space efficiently and in a parallelized fashion, while converging to a low cost function value. Three sets of couples of number of particles *N* and number of iterations *i* were tested. The nominal case *N* = 100; *i* = 10 represents a good balance between a large exploration of the search space and a reduced number of iterations for speed. We compared the obtained results against *N* = 50; *i* = 20 and *N* = 200; *i* = 5. The product *N* × *i* is kept constant in an effort to call the cost function evaluation a fixed number of times and therefore maintain similar computation times.

Furthermore, for the nominal case, we also evaluated the effect of the random component of the algorithm by running it three times with different random seeds.

## 5. Results

This section presents the experimental results obtained with the proposed algorithm. Different effects and situations as detailed in section 4 are explored in order to fully characterize the performance of the algorithm.

### 5.1. Qualitative Results

**Figure 8:**
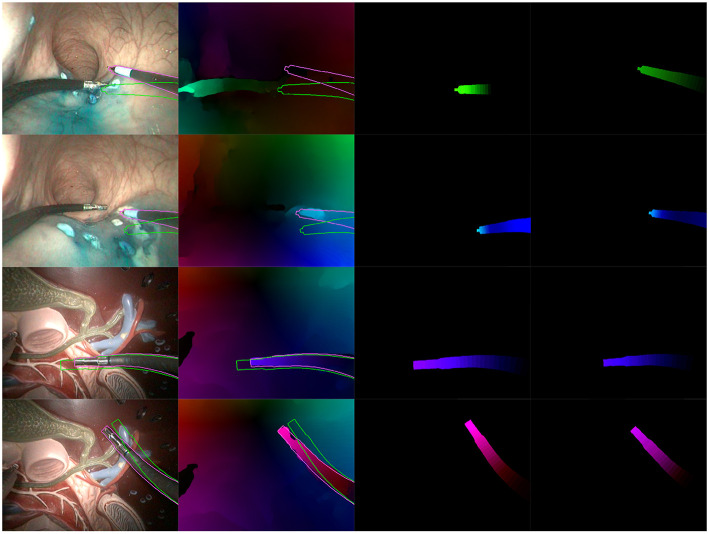
Examples of correct results obtained with our optimization algorithm. First two rows feature examples from the *in vivo* dataset, while the last two rows feature examples from the benchtop dataset. The columns are organized as follows, from left to right: Endoscopic image, with contours of the mask M before (green) and after (purple) optimization; Optical flow image $\hat{F}$ with the same superimposed contours; Virtual optical flow image F^*v*^ before optimization; Virtual optical flow image F^*v*^ after optimization.

**Figure 9:**
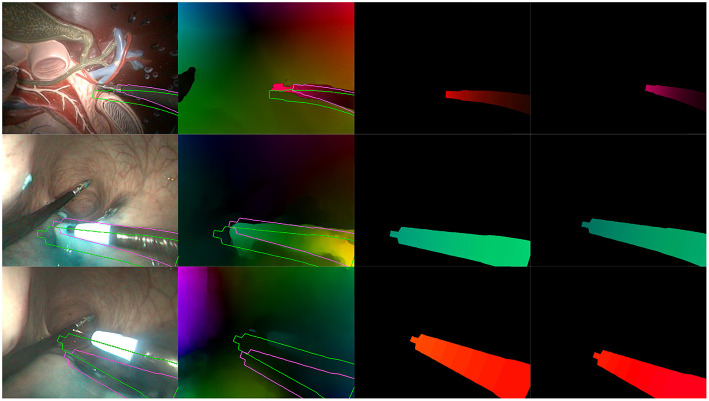
Examples of incorrect results obtained with our optimization algorithm. **(Top)** Incorrect extraction of the instrument tip. **(Middle)** Bad convergence due to a badly estimated $\hat{F}$. **(Bottom)** Incorrect estimation due to hand-eye calibration errors. Columns are the same as in Figure 8.

Figures 8, 9 present examples of optimization results obtained on both datasets (more examples provided in the Supplementary Material). Figure 8 features many examples where the optimized mask M almost perfectly matches with the contours of the instrument in the image, despite challenging conditions such as a very smooth optical flow around the instrument contour, and parts of the instrument looking very similar to its immediate surroundings [especially for the *in vivo* case, where the blue dye used during the surgical operation (Methylene blue) was very similar to the blueish hue of the instrument tip].

Figure 9 features a few examples of incorrect results after optimization. The top row shows a situation where the instrument tip has a quite different aspect from the rest of the instrument (in this case, the grasper has a metallic aspect while the body is covered by a black sheath). In this particular case, the image similarity metric *f*_*h*2_ in the cost function biased the search toward this result. The second row shows an example where optical flow estimations were made difficult by tool-tissue interactions as well as shadows from the instrument. As a result, the flow image $\hat{F}$ features a greenish area which is much larger than the tool area, making the flow similarity metrics *f*_*direct*_ and *f*_*h*1_ less specific. Finally, the bottom row on Figure 9 shows an example of incorrect result due to hand-eye calibration errors. Hand-eye calibration errors cannot always be compensated by modifying the robot pose parameters, especially when the robotic arms gets very far or very close from the camera. In the latter case, shown on Figure 9 in the bottom row, projection errors due to incorrect hand-eye calibration are amplified because the object is closer from the camera (i.e., a slight change of 3D position implies a large projection error in the image plane). For the presented case, errors are such that the corrections δ which could provide a qualitatively correct result are outside of the search space. As a result, the algorithm converges to a totally different pose. Note, however, that these results show optimizations performed on a single image; some of the incorrect results may be rejected by the consistency check.

### 5.2. Validation of the Cost Function

In order to evaluate the effectiveness of the proposed cost function independently of the convergence algorithm used, we looked at how the values of the cost function correlate with the Precision, Recall, and IoU metrics. For all the selected images in both datasets (with *S*_*rand*_ or *S*_*sel*_), we performed random modifications of δ around the nominal pose parameters *q*, and evaluated both the cost function and the corresponding Precision, Recall, and IoU. The results are a large sets of cost function evaluations, for various images coming from two datasets, in a variety of situations.

**Figure 10:**
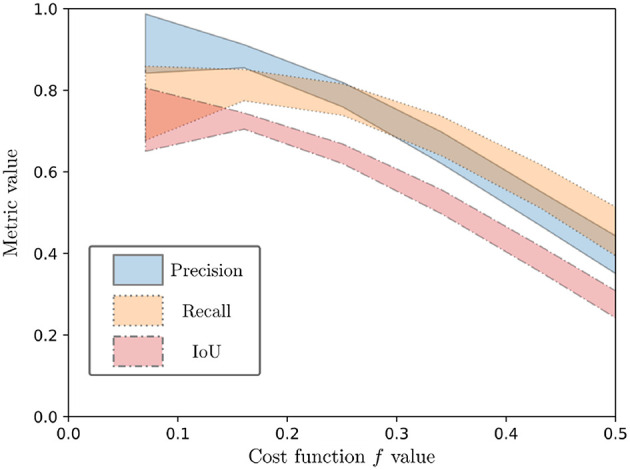
Ninety five percent confidence interval for a 5-order polynomial fitting of the performance metrics (Precision, Recall, and IoU) as a function of the evaluation function.

Spearman rank order correlation was used for estimating the correlation between the cost function and the metrics, since no linear correlation could be assumed *a priori*. The result is a high negative correlation of the cost function values with the considered metrics, with values of −0.67, −0.58, and −0.68 for correlation of f with the Precision, Recall and IoU metrics, respectively. This is illustrated on Figure 10, which shows the 95% confidence intervals after a 5-order polynomial fitting of the performance metrics with respect to the evaluation function. As can be seen from both Figure 10 and the Spearman rank correlation values, a diminution of the cost function is correlated with a positive variation of the metrics. Since all metrics represent best performance when they tend to 1, this shows the overall good performance of the proposed cost function.

### 5.3. Optimization Results

**Figure 11:**
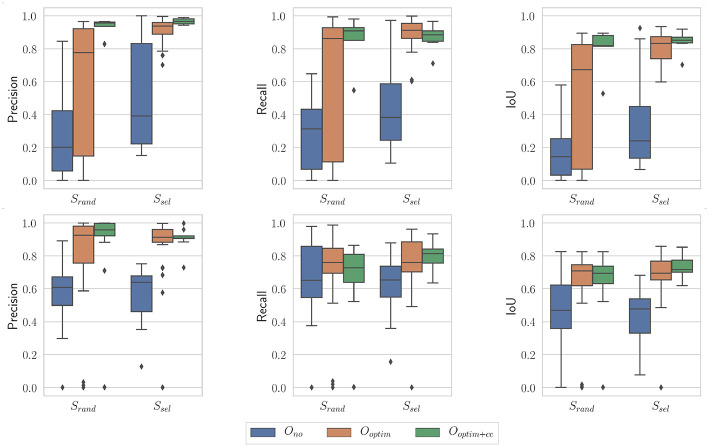
Boxplots showing the evaluation metrics for *N* = 100; *i* = 10. **(Top)** *In vivo* dataset; **(Bottom)** Benchtop dataset.

Figure 11 presents results obtained with the proposed algorithm for the nominal case (*N* = 100 and *i* = 10), on the benchtop and the *in vivo* datasets. A few observations can be made from these graphs. First, the raw kinematic model appears to provide poor quality outputs. This is all the more true on the *in vivo* dataset, which features ample tool-tissue interactions. Second, the optimization appears to provide a net increase in all metrics values (which was expected given results from section 5.2), while the consistency check gives an additional improvement to those values, especially in the *in vivo* case.

In order to quantitatively confirm these observations, we used statistical tests. After assessing the normality of the data, we performed a two-factor ANOVA. Factor *S* was the sampling strategy (*S*_*rand*_ or *S*_*sel*_), and factor *O* was the optimization type (*O*_*no*_, *O*_*optim*_, and *O*_*optim*+*cc*_). The effect of each factor was assessed. When statistical significance was found, *post-hoc* testing was performed using independent *t*-tests, with a Holm-Bonferroni correction in order to account for multiple testing. Finally, the size of the effect was also evaluated using the ω^2^ effect size measure (Ialongo, 2016).

On the *in vivo* dataset, the image sampling strategy *S* was found to have a significant influence on the results (*p* < 0.001 for all metrics), with a small effect size (ω^2^ ≃ 0.08 in all cases). Actually, one can see on Figure 11 that the metrics are slightly higher when using the image selection process than when using pure random sampling even for the unoptimized cases. The optimization type *O* was also highly significant for all metrics, with much larger effect sizes (see Table 1). *Post-hoc* testing confirmed that the optimization brings a clear improvement to the results, with both *O*_*optim*_ and *O*_*optim*+*cc*_ giving better results (i.e., higher metrics values on average), with high statistical significance (*p* < 0.001) when compared to the results obtained with *O*_*no*_. Although a clear trend can be seen on Figure 11, the consistency check did not provide a statistically significant improvement when combined with the image selection *S*_*sel*_ (*p* > 0.05 for the comparison of *O*_*optim*_ and *O*_*optim*+*cc*_ for the Precision, Recall, and IoU). When used with the random sampling strategy *S*_*rand*_, however, the consistency check brought higher Precision (*p* < 0.01) and IoU (*p* < 0.05) values. These results show that if the image selection procedure fails (for instance, if the kinematic model is too disturbed by tool-tissue interaction, or if no good images can be selected due to numerous direction changes in the motor input), the consistency check will help filtering out low quality optimization results.

**Table 1:** Summary of ANOVA results and effect size for optimization type factor *O*, for the considered metrics and the three sets of parameters for the PSO.

***In vivo*** **dataset**						
	**N = 50**		**N = 100**		**N = 200**	
	***p*****-value**	**ω**^**2**^	***p*****-value**	**ω**^**2**^	***p*****-value**	**ω**^**2**^
Precision	< 10^−5^	0.27	< 10^−5^	0.32	< 10^−5^	0.43
Recall	< 10^−5^	0.35	< 10^−5^	0.38	< 10^−4^	0.40
IoU	< 10^−5^	0.34	< 10^−5^	0.43	< 10^−5^	0.51
**Benchtop dataset**						
	**N = 50**		**N = 100**		**N = 200**	
	***p*****-value**	**ω**^**2**^	***p*****-value**	**ω**^**2**^	***p*****-value**	**ω**^**2**^
Precision	< 10^−5^	0.22	< 10^−5^	0.32	< 10^−5^	0.42
Recall	0.03	0.04	0.1	0.02	0.001	0.07
IoU	< 10^−5^	0.21	< 10^−5^	0.24	< 10^−5^	0.33

Results obtained on the benchtop dataset are less contrasted. One can see on Figure 11 that the image selection procedure *S*_*sel*_ has little effect on the metrics, which is confirmed by the ANOVA results: for the factor *S*, *p*-values are > 0.5 for all metrics, with a negative ω^2^ effect size (note that a negative effect size does not reflect a negative effect on the results, see Okada, 2017 for details). Actually, one can note that the results without optimization (*O*_*no*_) are much better than on the *in vivo* case. This is due to the fact that, although the robot was teleoperated, movements are overall smoother in this dataset, and tool-tissue interaction is not as intensive as in the *in vivo* case. These combined facts make the uncorrected kinematic model generally more correct than in the *in vivo* case, making the image selection procedure less critical for obtaining good results. On the other hand, the optimization factor provided statistically significant increases for the Precision and IoU metrics, with rather large effect sizes (see Table 1). For those metrics, *post-hoc* testing showed that both *O*_*optim*_ and *O*_*optim*+*cc*_ gave better results (i.e., higher metrics values on average), with high statistical significance (*p* < 0.001) when compared to the results obtained without optimization (*O*_*no*_), when used with either *S*_*sel*_ or *S*_*rand*_. The effect of the consistency check was not found to be significant when comparing *O*_*optim*_ and *O*_*optim*+*cc*_ with *post-hoc* testing. In fact, in this dataset, ample movement of the robot arm, combined with ample and constant movement of the background, make the flow images $\hat{F}$ easier to estimate for the FlowNet algorithm. The smoother motion also makes the differential kinematics less erroneous. Those two combined factors reduce the effect of the consistency check although a small trend toward an increase of Precision and IoU can be observed on Figure 11.

**Figure 12:**
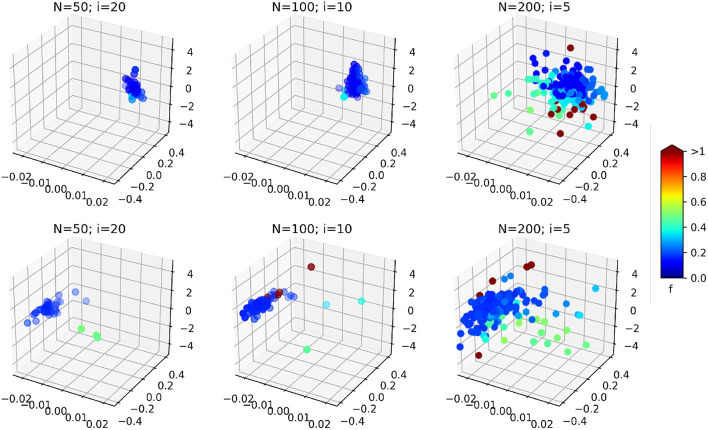
3D scatter plots representing the position of the swarm particles in the search space at the end of the optimization (on a single image, without consistency check), for the different PSO parameters. The colors represent associated cost function values. **(Top)** *In vivo* dataset; **(Bottom)** Benchtop dataset. The three axes are the three components of δ: the bending curvature [*m*^−1^], the robot rotation [*rad*], and the robot insertion [*mm*].

Another interesting effect can be seen from Table 1: the increase of the PSO parameters *N* seems to increase the effect of the optimization (effect size of the optimization type factor *O* in the ANOVA). As it is illustrated on Figure 12, the resulting swarms at the end of optimization seem to all converge to the same result, but with a higher density of low cost-function particles in the case *N* = 200; *i* = 5, leading perhaps to better results in this case. This result should however be weighed against the average optimization time per image. As explained in section 4.3.3, the product *N* × *i* was kept constant in order to obtain similar computation times for all cases. In practice, the optimization times obtained per images (average ± standard deviation) are:

- 9.3 ± 3.8 seconds for *N* = 50; *i* = 20
- 12.2 ± 4.4 seconds for *N* = 100; *i* = 10
- 15.8 ± 4.3 seconds for *N* = 200; *i* = 5

It can be observed that increasing the total number of particles also increases the computation time, even though the number of iterations is reduced. In fact, the product *N* × *i* gives the total *maximum* number of cost function evaluations. When a smaller swarm is used with an increased number of iterations, the particles will likely converge before the prescribed total number of iterations, causing the PSO algorithm to terminate with a lower number of cost function evaluations. In our case, the *N* = 50; *i* = 20 case provides a 60% speedup over the *N* = 200; *i* = 5 case. The case *N* = 100; *i* = 10 represents a good compromise between an increased effect size of the optimization (see Table 1) and a reduced overall computational time.

**Figure 13:**
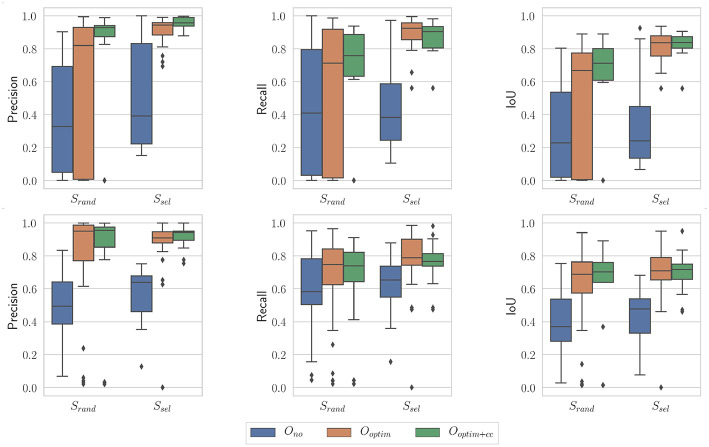
Boxplots showing the evaluation metrics for *N* = 100; *i* = 10. **(Top)** *In vivo* dataset; **(Bottom)** Benchtop dataset. The random seed used is different from the one in Figure 11.

Finally, the influence of the stochastic essence of the PSO algorithm was assessed by running the algorithm multiple times on the same images with different random seeds. An example of optimization results is shown on Figure 13. The general trend is the same as the case presented on Figure 11. For the non-optimized case *O*_*no*_, one can note that the randomly selected images perform poorly in this case (especially for the *in vivo* case), which is a byproduct of the random sampling. The *S*_*sel*_ case is perfectly identical to the case presented on Figure 11, since the image selection procedure does not feature any random component. Results obtained with optimization, with or without the consistency check, are not statistically significantly different between the results presented on Figure 13 and the ones presented on Figure 11 (*p* > 0.05 for all combinations of *S*_*rand*_ and *S*_*sel*_ on one hand, and of *O*_*optim*_ and *O*_*optim*+*cc*_ on the other hand). These results show that our proposed algorithm is only marginally affected by the stochastic components in the PSO algorithm.

## 6. Discussion and Conclusion

In this paper, we presented a novel method for automatic labeling of robotic instruments in endoscopic images. The proposed method is compatible with both rigid-body tools (e.g., robotic laparoscopy tools) and continuum robots (e.g., flexible endoscopy tools), provided that joint values can be obtained, and that a kinematic model of the robot is available. Indeed, the proposed algorithm makes use of the robot's forward and differential kinematics, together with image and optical flow measurements, in order to optimize a cost function over the robot pose parameters. The resulting mask of the robot shape projected on the image gives a pixel-wise binary classification of the robot in the endoscopic image. A consistency check procedure after the optimization was also proposed in order to reject outliers. The proposed algorithm is particularly interesting in two aspects. First, no human intervention is required, in opposition to the tedious process of manual image annotation and labeling. Second, the use of optical flow makes the overall algorithm independent of the visual aspect of the robot. This point is particularly important since the aspect of both the instrument and the environment may change during the surgery, for instance due to smoke or changing lighting conditions.

Validation was performed using a flexible robotic endoscopy platform, with cable-actuated flexible endoscopic tools. Images were acquired in a benchtop setting with phantoms of the human digestive system, and in an *in vivo* setting during a colorectal Endoscopic Submucosal Dissection performed on a porcine model. The parameters of the algorithm were extensively studied in order to characterize the behavior and performance of the algorithm. Results show that the proposed algorithm is robust, and allows providing good quality labels in challenging conditions.

Nevertheless, the study has limitations which should be acknowledged. First, the labels obtained using our method have a reasonably good quality, with an IoU reaching high levels especially in the *in vivo* images. Perfect labels are however not obtained, for various reasons related to incorrect optimization results not being rejected by the consistency check, or incorrect kinematic model assumptions (in our study we used the constant curvature assumption, which is an approximation). The obtained noisy labels could be combined with approaches robust to label noise, for instance random forests using bootstrap aggregating (Breiman, 1996), or more recent neural network-based approaches (Reed et al., 2014). A future, related direction of work could be to run our algorithm on various *in vivo* datasets, in order to be able to infer the average noise distribution in the labels obtained, in combination with approaches such as (Sukhbaatar and Fergus, 2014).

Another limitation to the present study is the fact that validation was performed on a limited number of images. This is in part due to the inherent difficulty and ethical questions raised when organizing multiple experiments on animal models. The limited number of images is however not really a problem for the intended use of our algorithm. In fact, the main interesting use of the algorithm could be for online classifier training, during a given surgery. In this use case, labels would be extracted on the fly as the surgery evolves, in order to train or fine-tune a classifier for segmenting the tool. Extracting a few, relevant and high quality labels using our approach would, in this case be required to train a surgery-specific classifier. Having only a few labeled examples usually causes the classifier to overfit on the training data, which would not be a problem in this case since it would be highly correlated to the data used for subsequent inference queries. In this context, lightweight classifiers such as the ones used in Garćıa-Peraza-Herrera et al. (2016); Rocha et al. (2019) could be interesting to use. It should be noted that this use case requires being able to run our algorithm substantially faster than in the present study, which we believe is achievable thanks to careful code optimization.

Finally, several other aspect of the algorithm may also be improved in order to produce higher quality results. The consistency check procedure could be made more robust by considering more than two consecutive images, for instance in a Bayesian Recursive Filtering approach (e.g., using a particle filter) provided, once again, that the optimization time can be reduced. Another interesting future line of work could be to consider the uncertainty in the optical flow estimates in the cost function as a weight, using for instance an approach such as (Ilg et al., 2018). Finally, we will investigate how full pose estimation could be achieved by combining the above-mentioned techniques as well as adding the hand-eye calibration parameters in the optimization variables. This last point is especially challenging since 3D pose estimation is hard to retrieve from 2D images, but we believe that the information added by the differential kinematics on one hand and the optical flow on the other hand could help reduce this uncertainty.

## Data Availability

The datasets generated for this study are available on request to the corresponding author.

## Ethics Statement

The study protocol for this experiment was approved by the Institutional Ethical Committee on Animal Experimentation (ICOMETH No. 38.2011.01.018). Animals were managed in accordance with French laws for animal use and care as well as with the European Community Council directive no. 2010/63/EU.

## Author Contributions

BR, VB, and FN contributed to the design of the algorithms and their implementation, acquired the experimental data, and edited the manuscript. BR and FN generated the experimental results and prepared the manuscript and the figures.

### Conflict of Interest Statement

The authors declare that the research was conducted in the absence of any commercial or financial relationships that could be construed as a potential conflict of interest.

## Supplementary Material

A Zip archive is attached to this publication as supplementary material. It includes 29 exemples of optimization results in the format of TIFF images, for both datasets. A text readme is included, which explains the data format and contents in details.
