# Supplementary material for: Combining Differential Kinematics and Optical Flow for Automatic Labeling of Continuum Robots in Minimally Invasive Surgery
Source: Front Robot AI. 2019 Sep 6;6:86. doi: 10.3389/frobt.2019.00086 (PMC7805658; doi:10.3389/frobt.2019.00086)
Supplement: Supplementary file 1 [file Presentation_1.pdf]

Appendix to *Combining differential kinematics and optical flow for automatic labelling of continuum robots in minimally invasive surgery*

Benoît Rosa *, Valentin Bordoux, and Florent Nageotte

ICube, CNRS, University of Strasbourg, INSA Strasbourg, France

Correspondence*:
Benoît Rosa
b.rosa@unistra.fr

This document is an Appendix to the following paper :

B. Rosa, V. Bordoux, and F. Nageotte, *Combining differential kinematics and optical flow for automatic labelling of continuum robots in minimally invasive surgery*, Frontiers in Robotics & AI, DOI: 10.3389/frobt.2019.00086

APPENDIX: KINEMATIC MODEL EQUATIONS FOR THE STRAS ROBOT

This appendix details the kinematic model equations for the STRAS robot used in this study. The robot is composed of a main arm carrying two flexible robotic arms. The kinematic model of the flexible arms (named robot in the following) is described below.

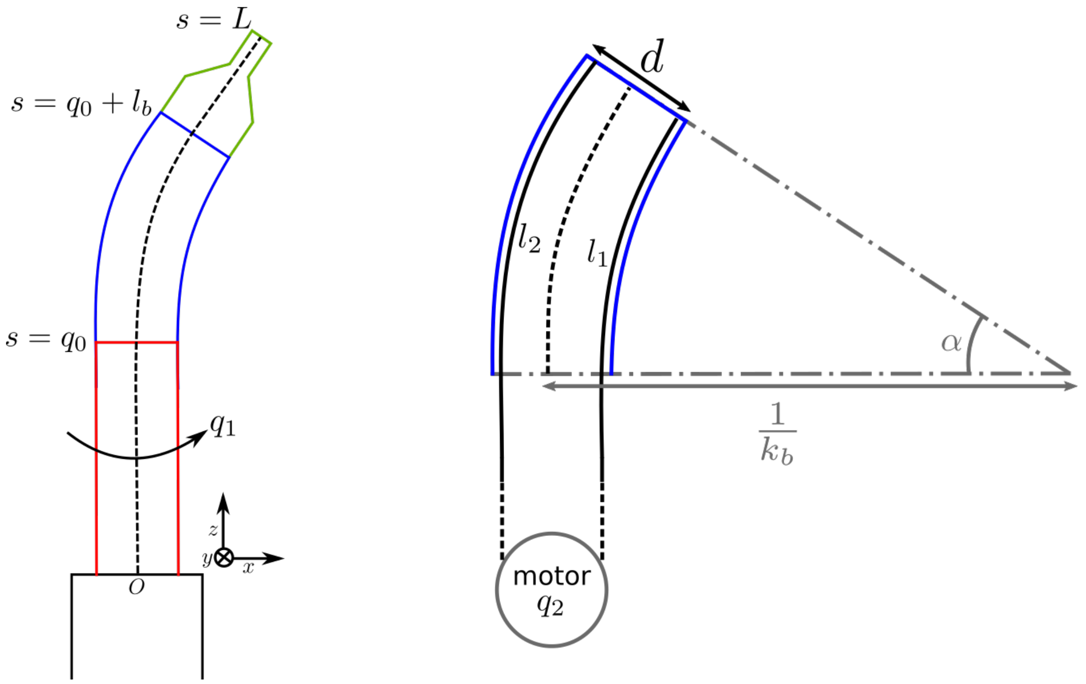

Figure 1. Schematic description of a robotic flexible arm and its kinematics. Left: general schematic showing the robot and its three sections. Right: zoom on the bending section

The robot is composed of three sections, as depicted on Fig. 1. We make here the assumption that the bending section (in blue) has a constant curvature radius, while the two other sections (in red and in green) are straight sections. We then use the formalism presented in Webster and Jones (2010) in order to compute the complete kinematic model of the robot. The kinematic model is expressed in the robot base frame r , which is depicted as an xyz triplet with origin O on Fig. 1.

The first section (in red on Fig. 1) is a simple straight section. The joint variable q_0 is obtained from the first motor encoders, and controls the insertion length of the robot. It is calibrated in such a way that the length of the first section is equal to q_0. Moreover, the base of the robot is rotated by the second motor. The joint variable q_1 is the rotation angle of the robot base around the vector z. By convention, q_1 is equal to 0 when the bending plane is in the xz plane of the base frame (situation depicted on Fig. 1). The combination of the two above-mentioned transformations writes :

$$\forall s \in [0, q_0], g(q, s) = \begin{pmatrix} \cos(q_1) & -\sin(q_1) & 0 & 0 \\ \sin(q_1) & \cos(q_1) & 0 & 0 \\ 0 & 0 & 1 & s \\ 0 & 0 & 0 & 1 \end{pmatrix} \tag{1}$$

The second section (in blue on Fig. 1) is a bendable section, whose bending is controlled by two cables attached on its inner sides. A motor controls the differential length of the cables, giving the joint variable :

$$q_2 = l_2 - l_1 \tag{2}$$

Following the constant curvature assumption, one can write

$$l_b = \frac{\alpha}{k_b}, \tag{3}$$

where l_b is the bending section's length, α its bending angle, and k_b the curvature of the section. Considering that the diameter d of the section is known, one can also write :

$$l_1 = \alpha(\frac{1}{k_b} - \frac{d}{2}) \tag{4}$$

$$l_2 = \alpha(\frac{1}{k_b} + \frac{d}{2}) \tag{5}$$

Combining equations 2 – 5, we obtain:

$$k_b = \frac{q_2}{d\, l_b} \tag{6}$$

Using the formalism from Webster and Jones (2010), we define ξ as $\begin{bmatrix} 0 & 0 & 1 & 0 & k_b & 0 \end{bmatrix}^T$. Taking the matrix exponential of the skew-symmetric matrix $\hat{\xi}$ associated with ξ, we obtain:

$$\forall s \in [q_0, q_0 + l_b], g(q, s) = g(q, q_0)e^{(\hat{\xi}(s-q_0))} \tag{7}$$

Finally, the last section of the robot arm (in green on Fig. 1) is again a simple straight section. It is then straightforward to write:

$$\forall s \in [q_0 + l_b, L], g(q, s) = g(q, q_0 + l_b) \begin{pmatrix} 1 & 0 & 0 & 0 \\ 0 & 1 & 0 & 0 \\ 0 & 0 & 1 & (s - q_0 - l_b) \\ 0 & 0 & 0 & 1 \end{pmatrix} \tag{8}$$

This appendix demonstrates how the whole kinematic model is obtained for any $s \in [0, L]$, given the robot joint variables $q = [q_0, q_1, q_2]$ and its geometric properties (length of the sections and diameter of the bending section). The robot Jacobian $J(q, s)$ is obtained by differentiating the above equations with respect to q_0, q_1, and q_2.

REFERENCES

Webster, R. J. and Jones, B. A. (2010). Design and Kinematic Modeling of Constant Curvature Continuum Robots: A Review. *The International Journal of Robotics Research* 29, 1661–1683. doi:10.1177/0278364910368147
