# Supplementary figures and images for: Combining Differential Kinematics and Optical Flow for Automatic Labeling of Continuum Robots in Minimally Invasive Surgery
Source: Front Robot AI. 2019 Sep 6;6:86. doi: 10.3389/frobt.2019.00086 (PMC7805658; doi:10.3389/frobt.2019.00086)

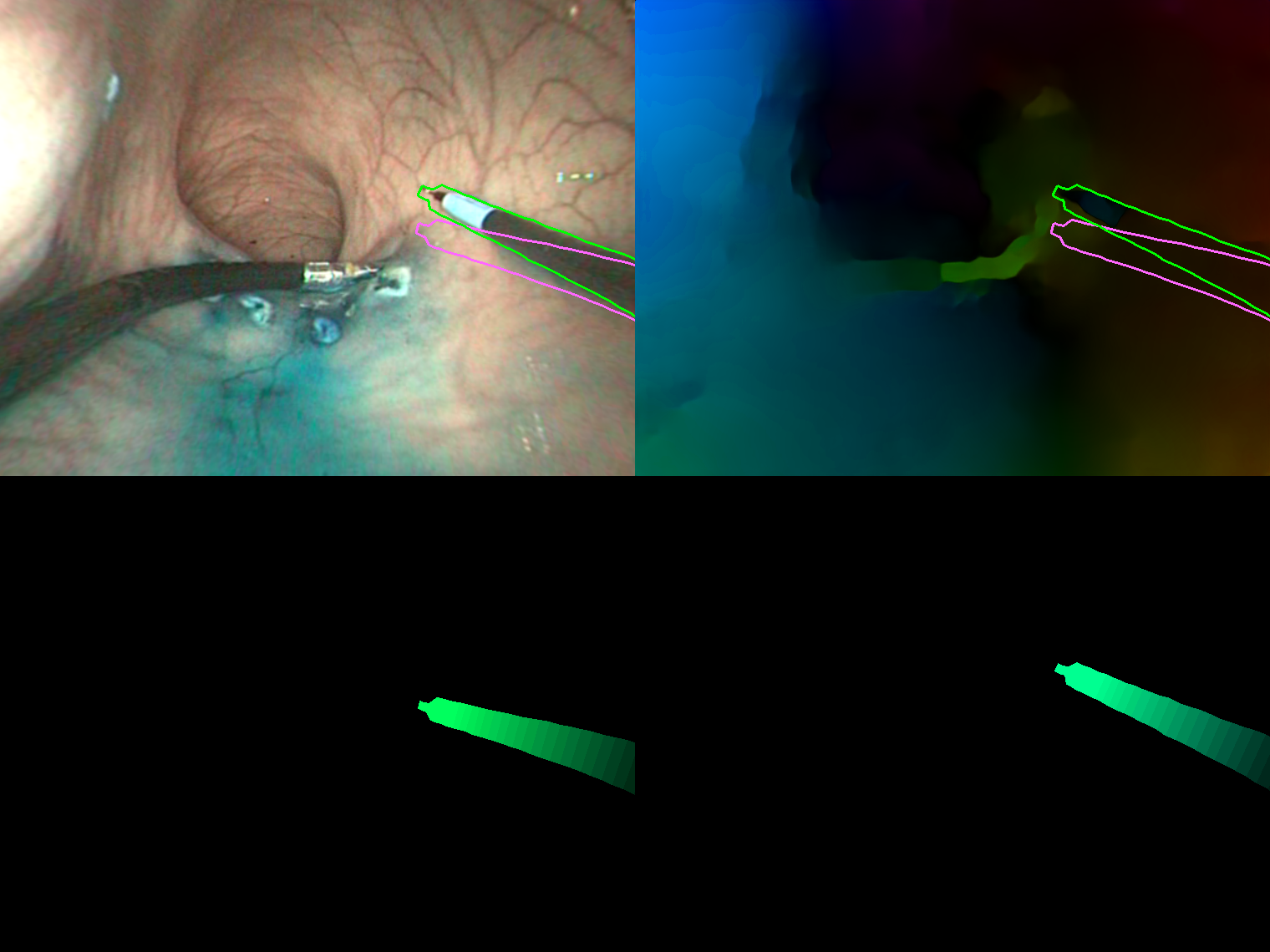

Supplement: Supplementary file 2 [file Data_Sheet_1.ZIP › 10_complete.tiff]

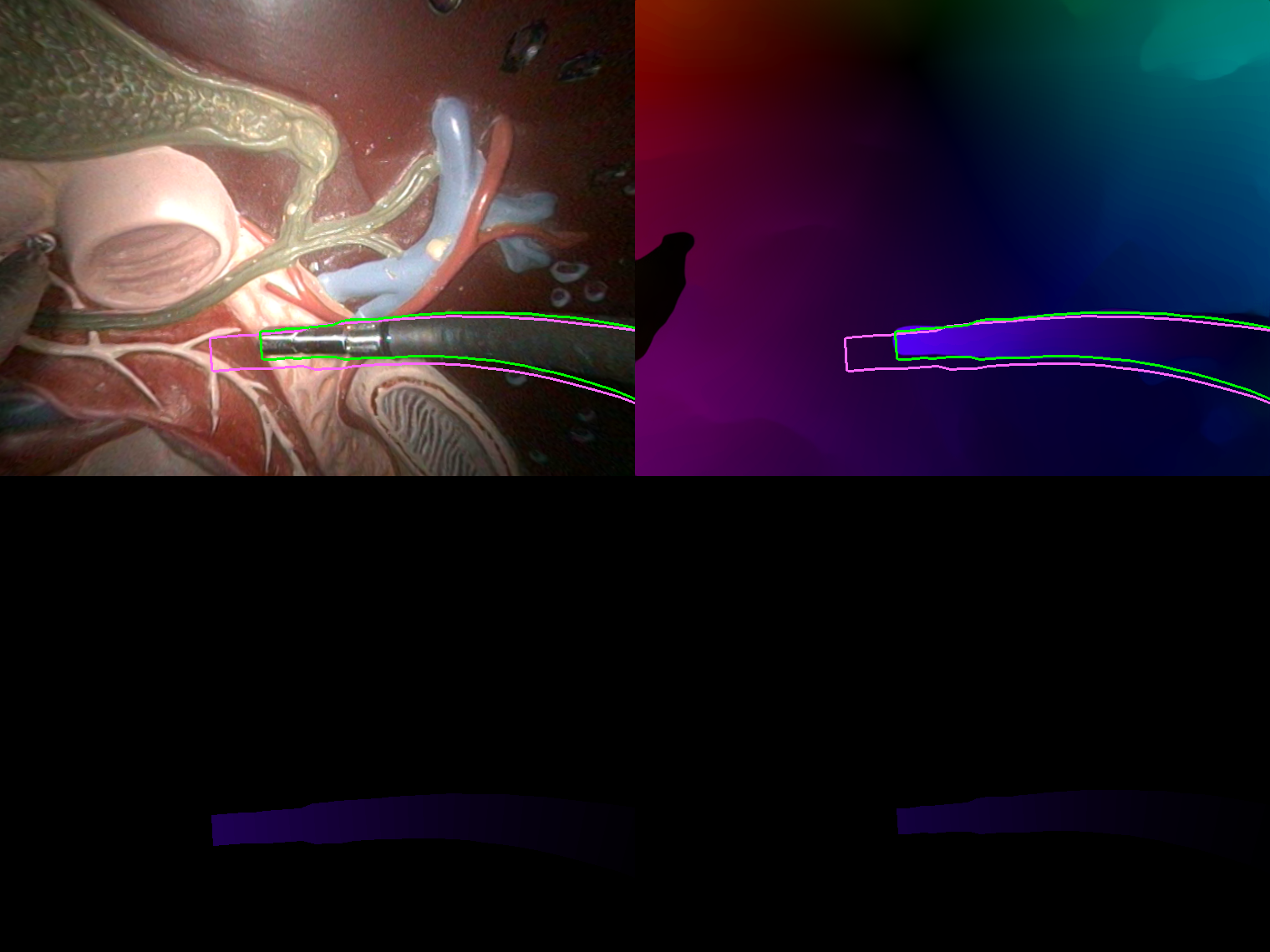

Supplement: Supplementary file 2 [file Data_Sheet_1.ZIP › 11_complete.tiff]

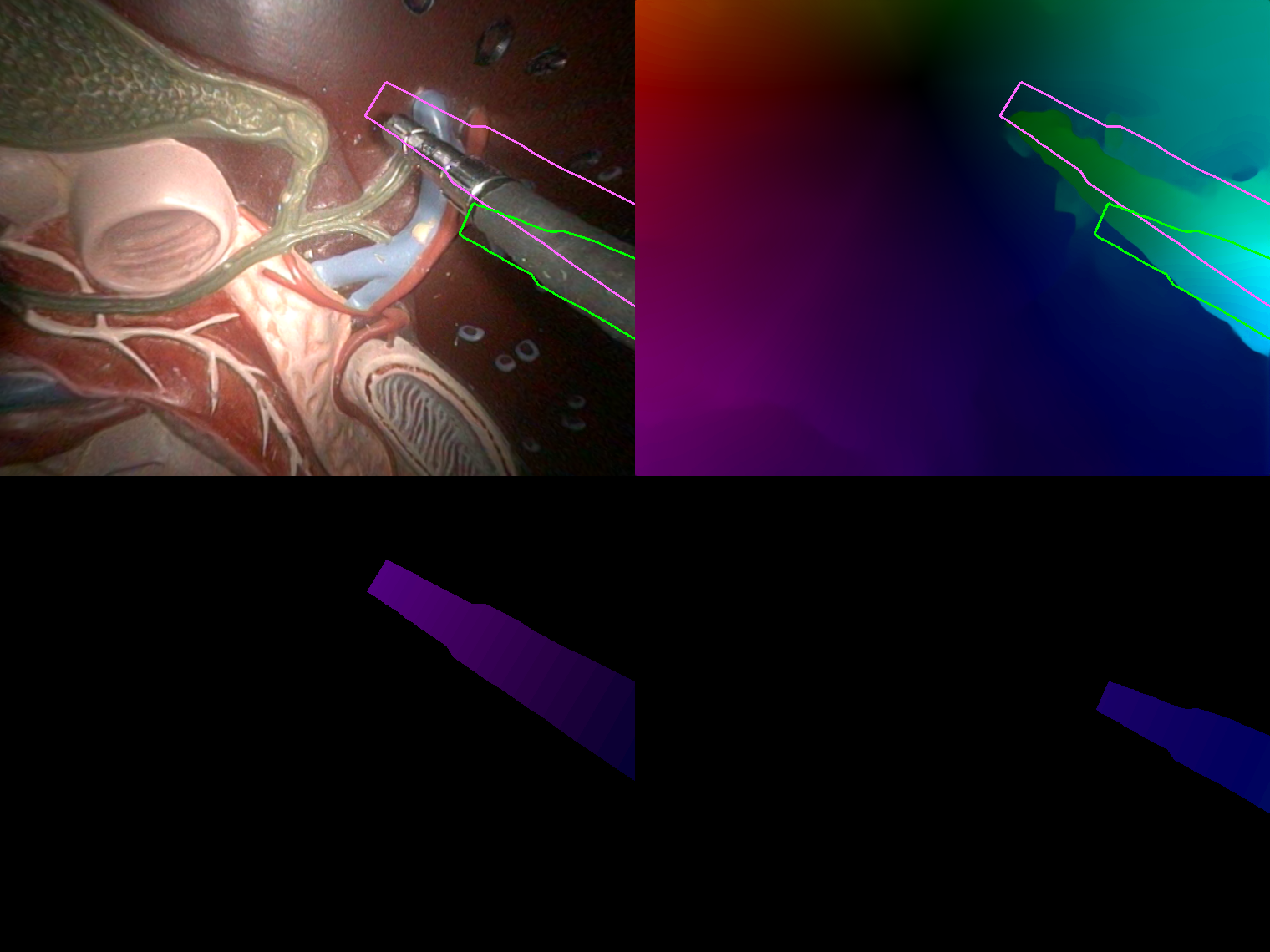

Supplement: Supplementary file 2 [file Data_Sheet_1.ZIP › 12_complete.tiff]

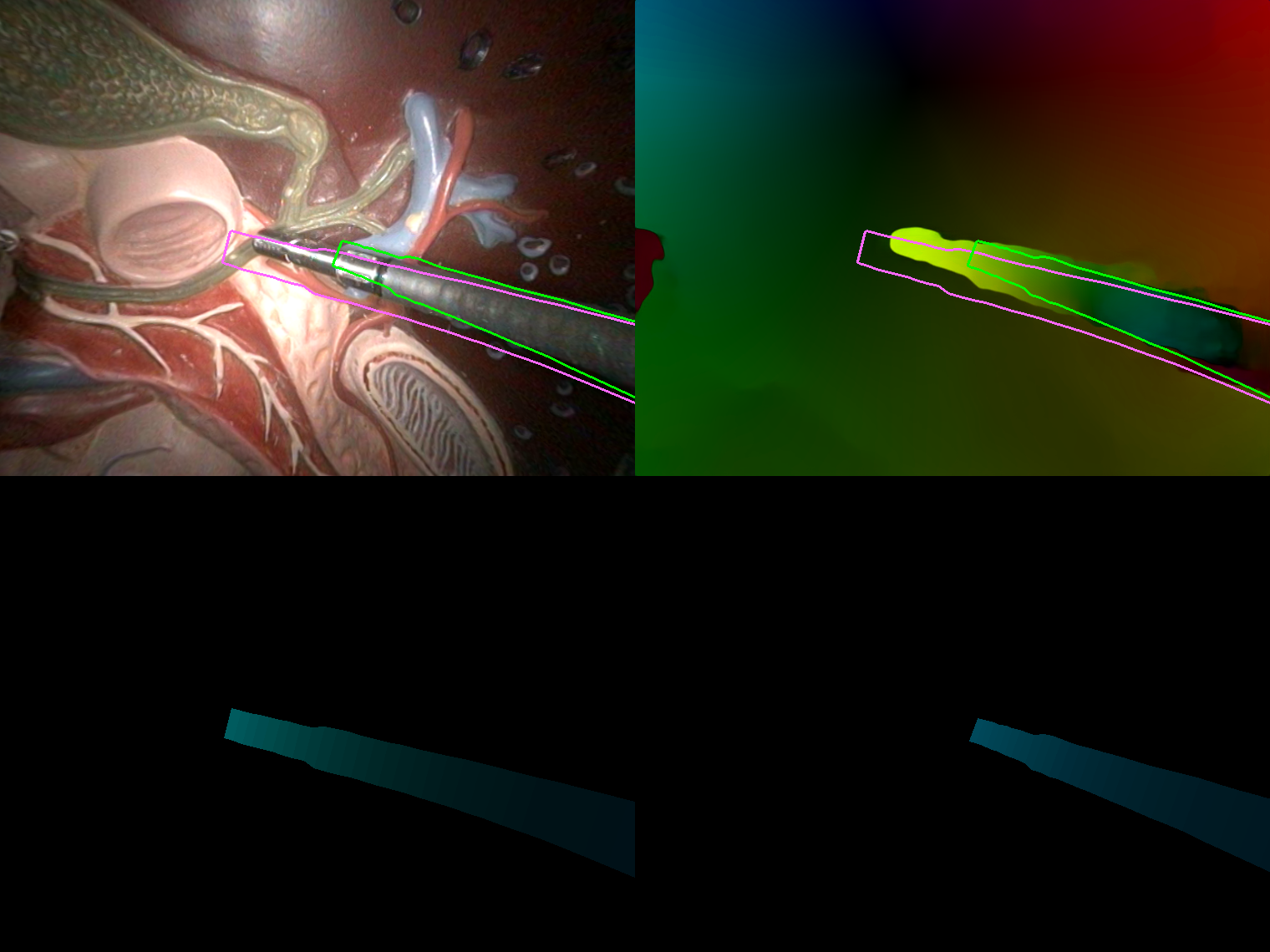

Supplement: Supplementary file 2 [file Data_Sheet_1.ZIP › 13_complete.tiff]

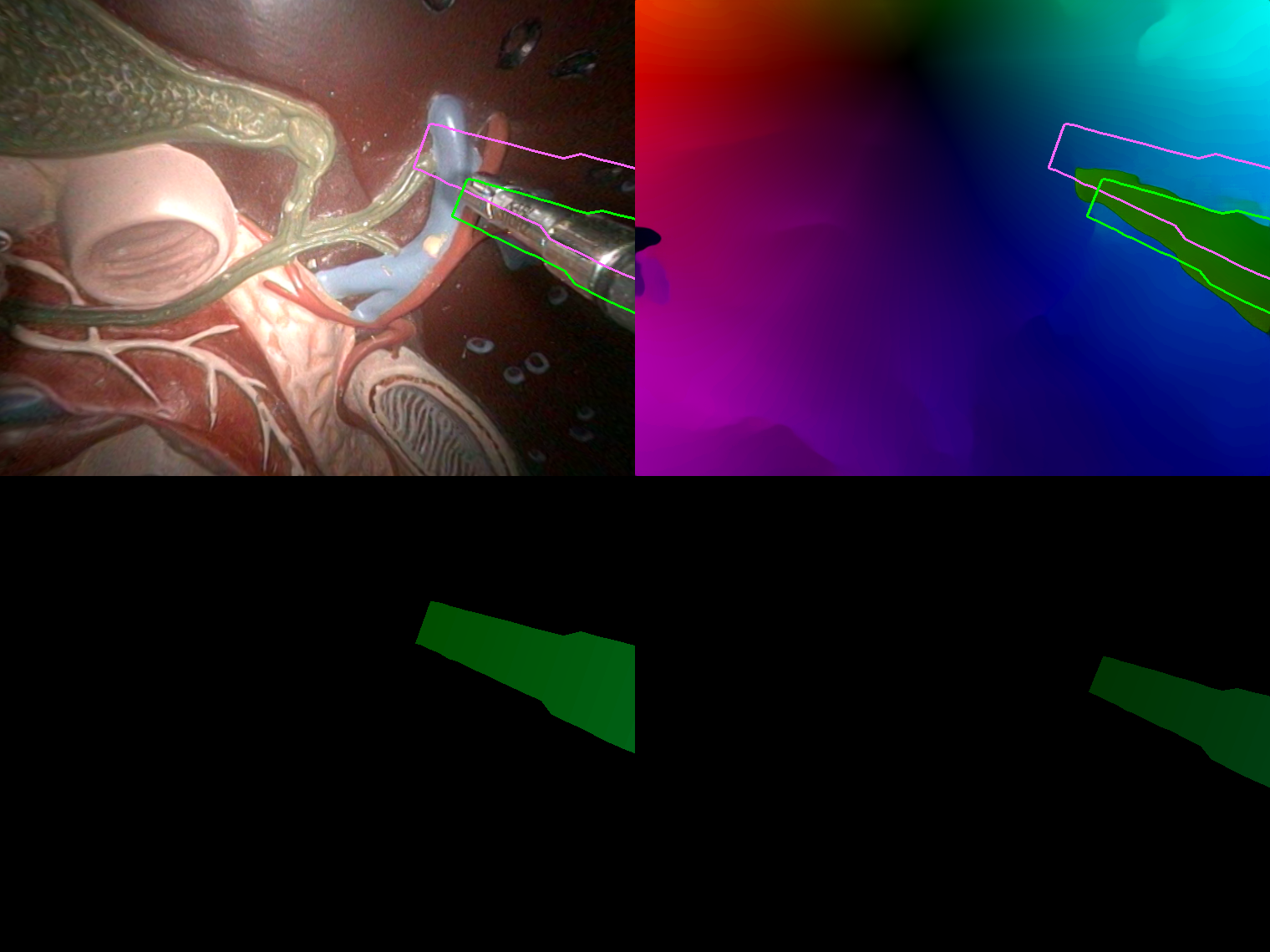

Supplement: Supplementary file 2 [file Data_Sheet_1.ZIP › 14_complete.tiff]

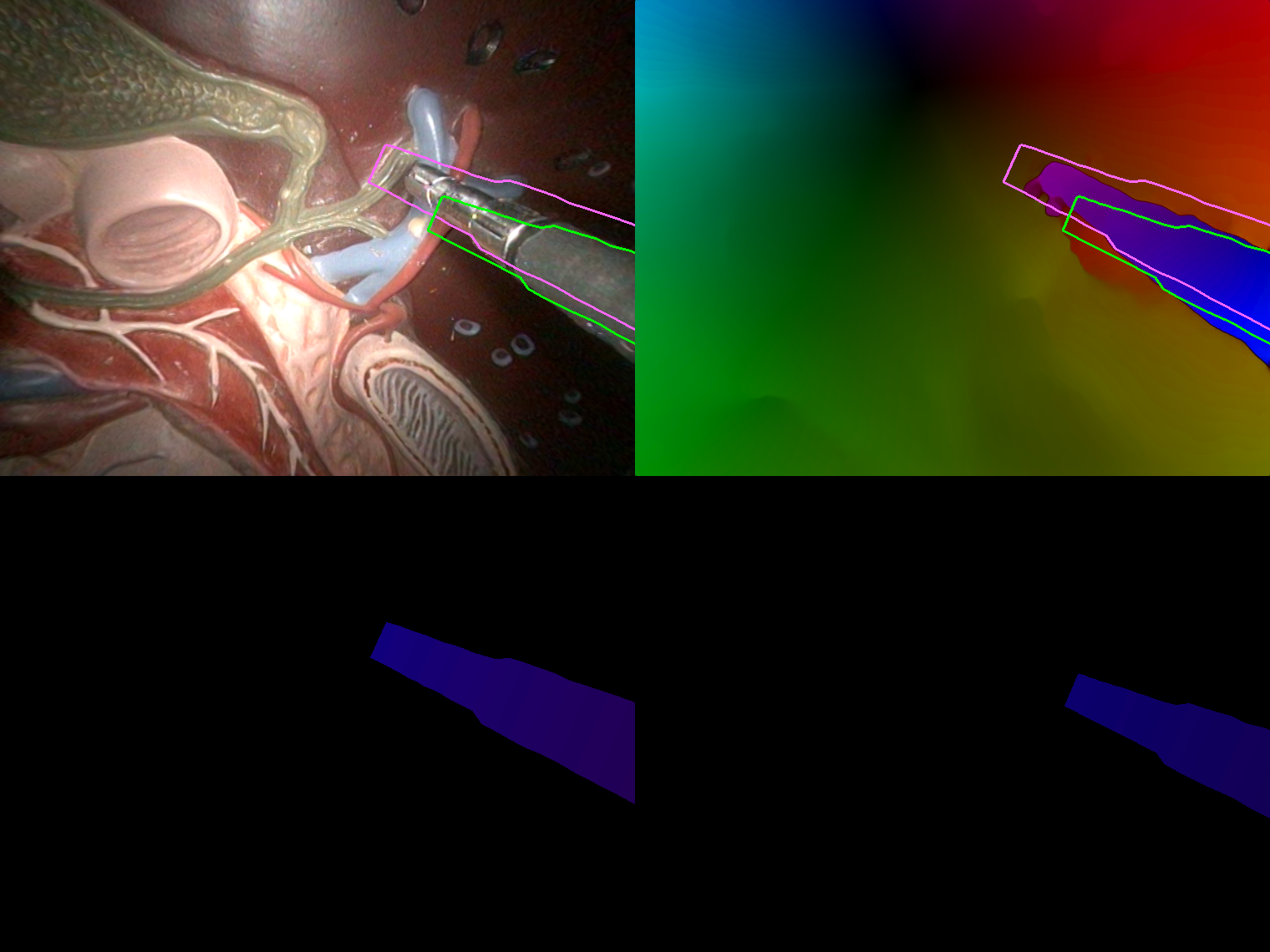

Supplement: Supplementary file 2 [file Data_Sheet_1.ZIP › 15_complete.tiff]

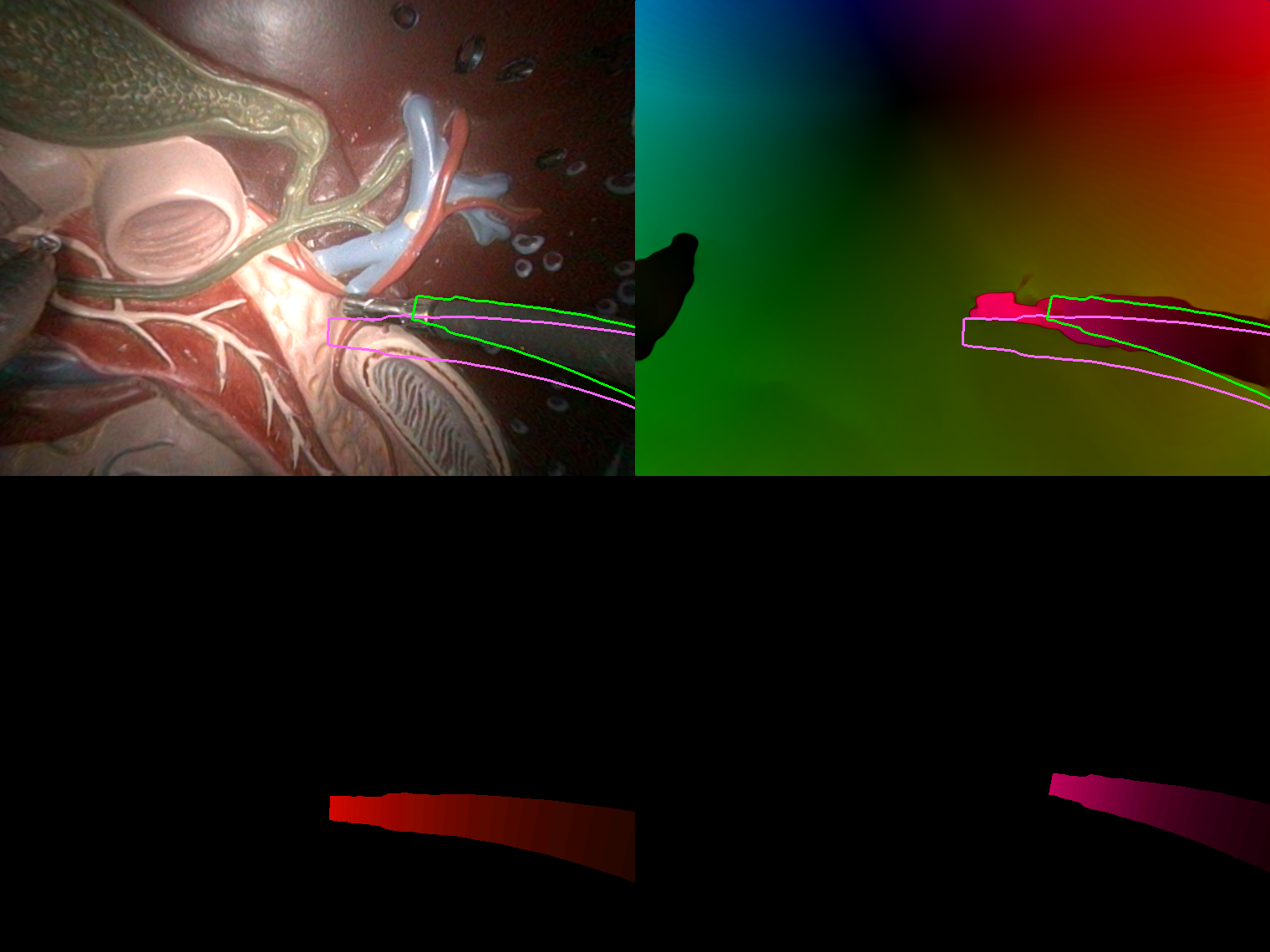

Supplement: Supplementary file 2 [file Data_Sheet_1.ZIP › 16_complete.tiff]

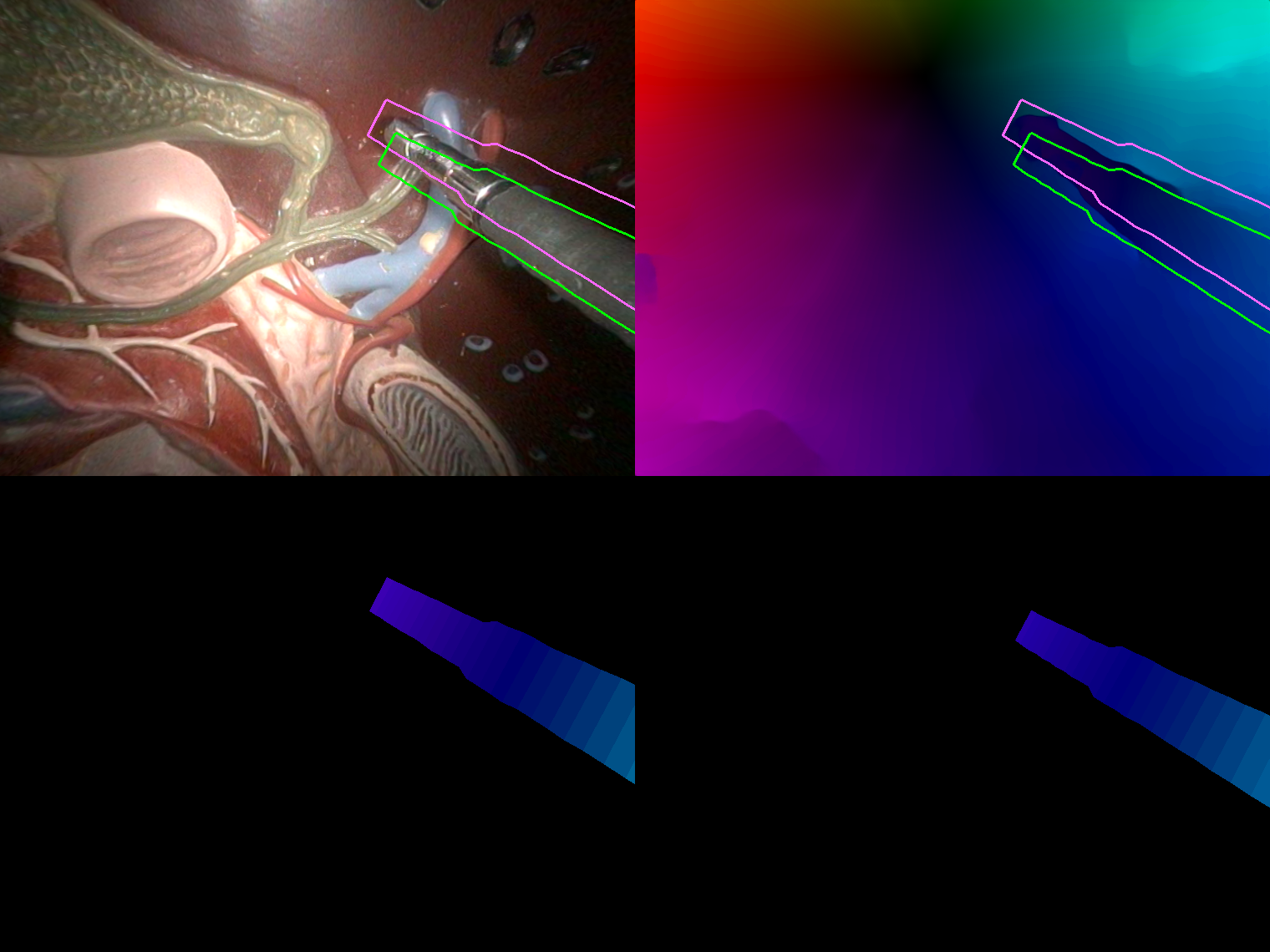

Supplement: Supplementary file 2 [file Data_Sheet_1.ZIP › 17_complete.tiff]

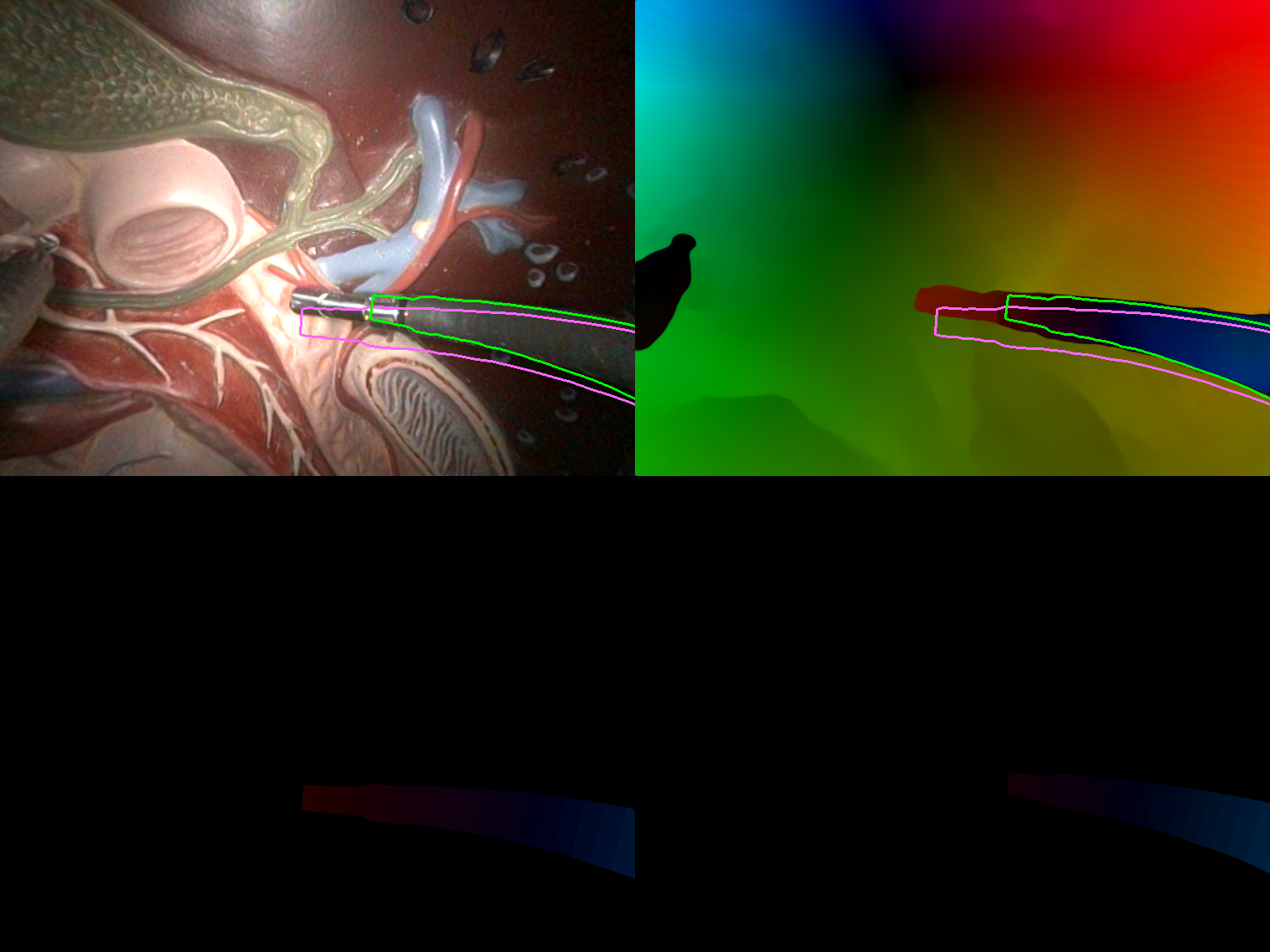

Supplement: Supplementary file 2 [file Data_Sheet_1.ZIP › 18_complete.tiff]

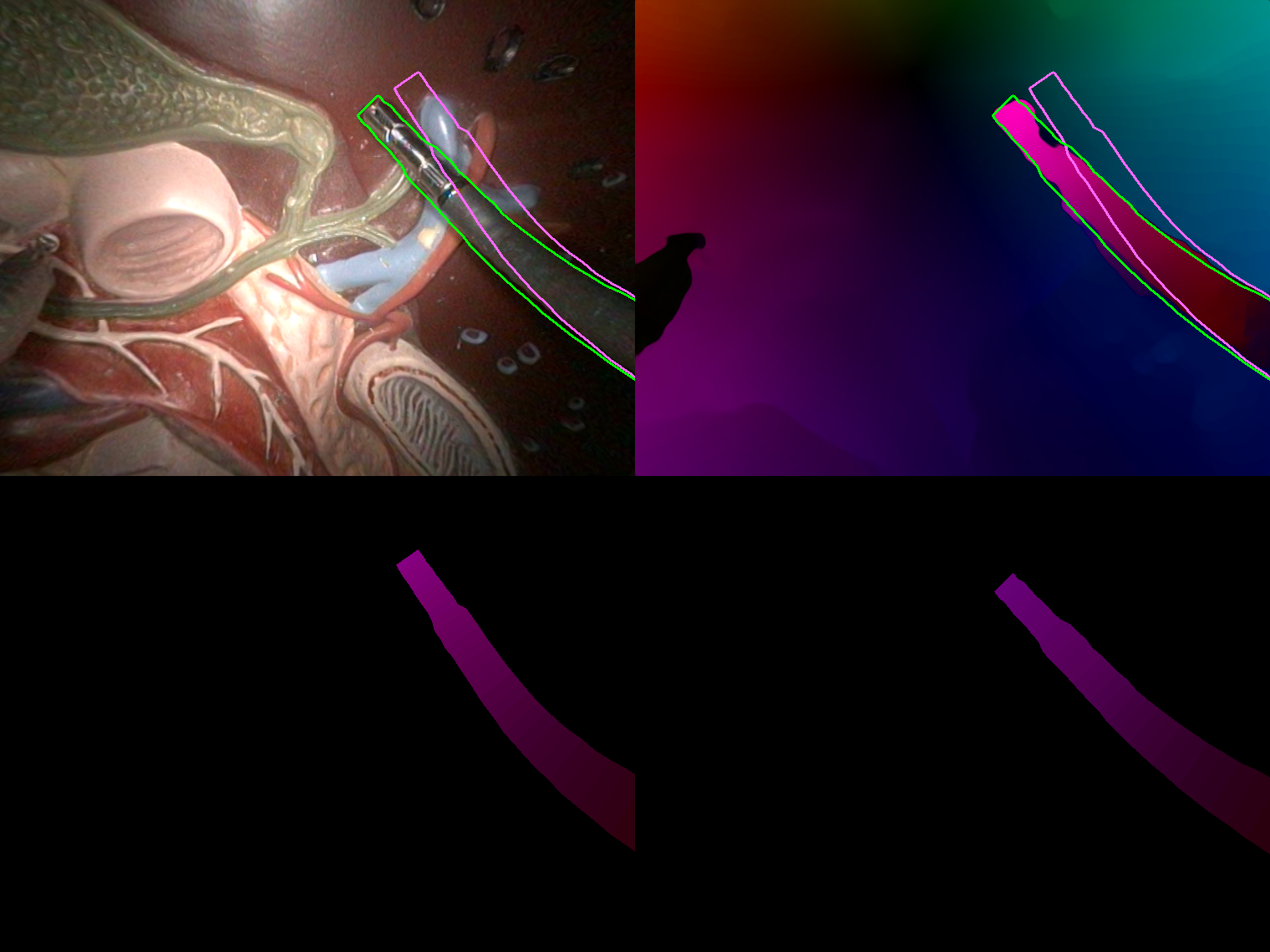

Supplement: Supplementary file 2 [file Data_Sheet_1.ZIP › 19_complete.tiff]

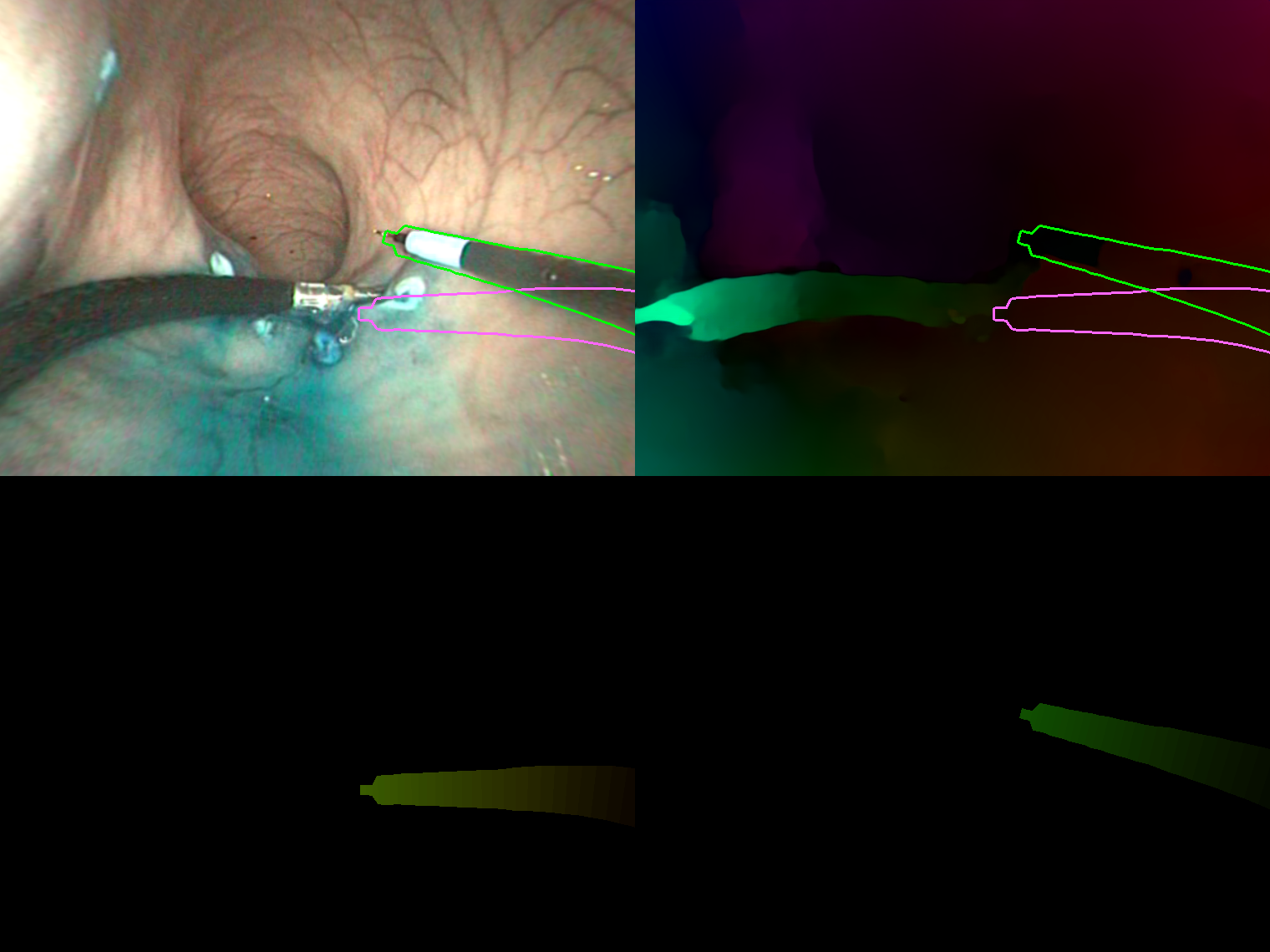

Supplement: Supplementary file 2 [file Data_Sheet_1.ZIP › 1_complete.tiff]

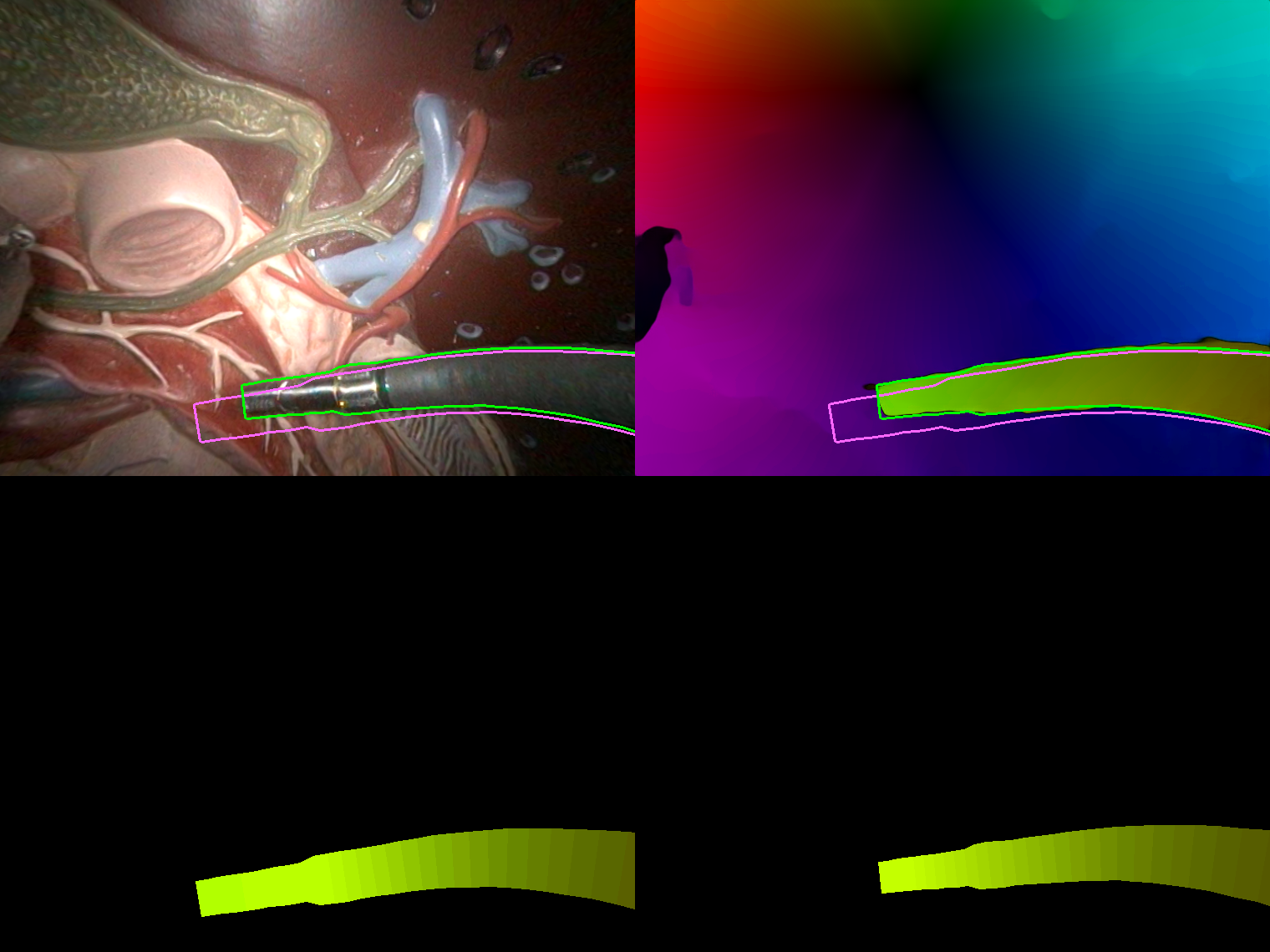

Supplement: Supplementary file 2 [file Data_Sheet_1.ZIP › 20_complete.tiff]

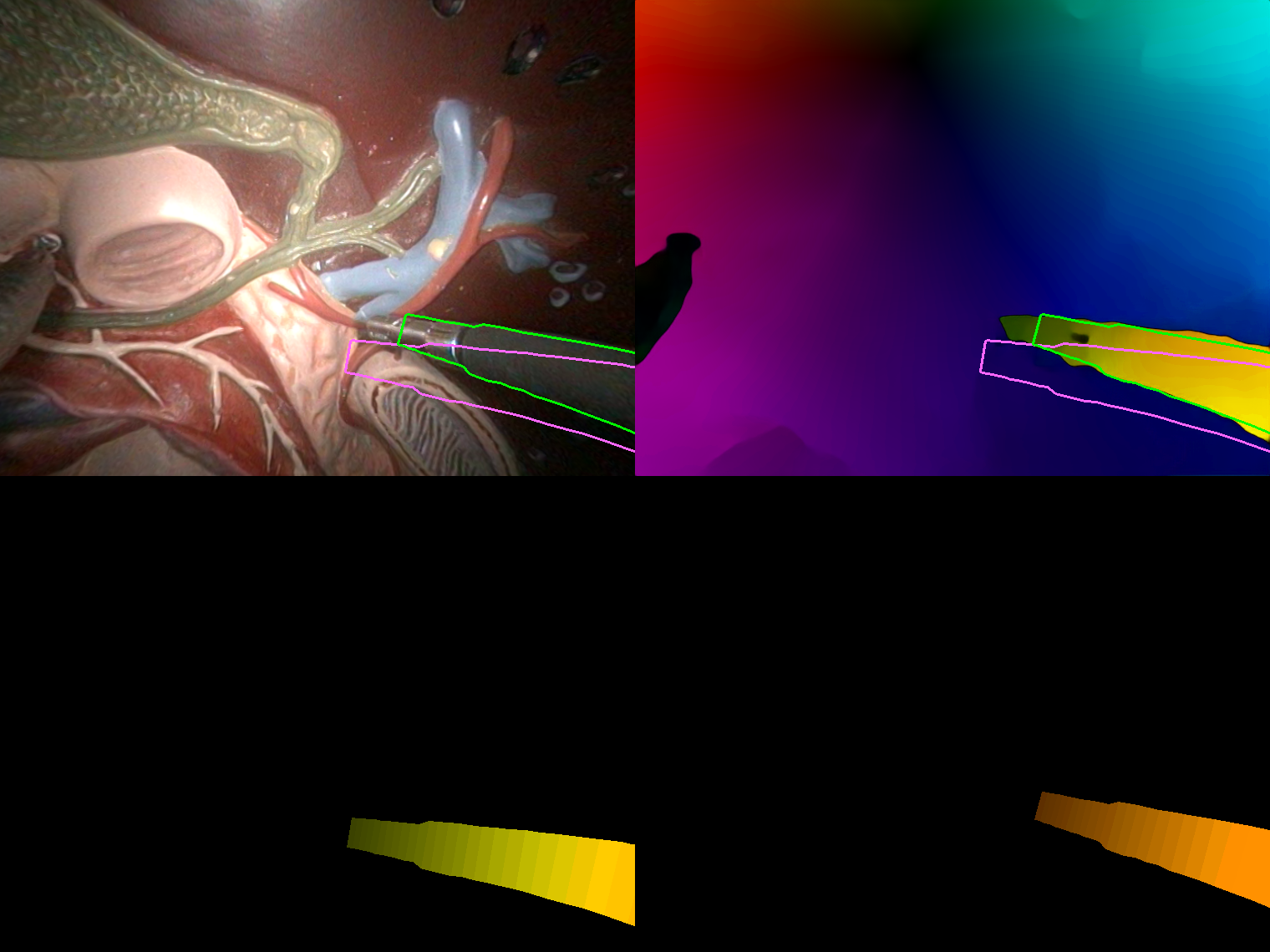

Supplement: Supplementary file 2 [file Data_Sheet_1.ZIP › 21_complete.tiff]

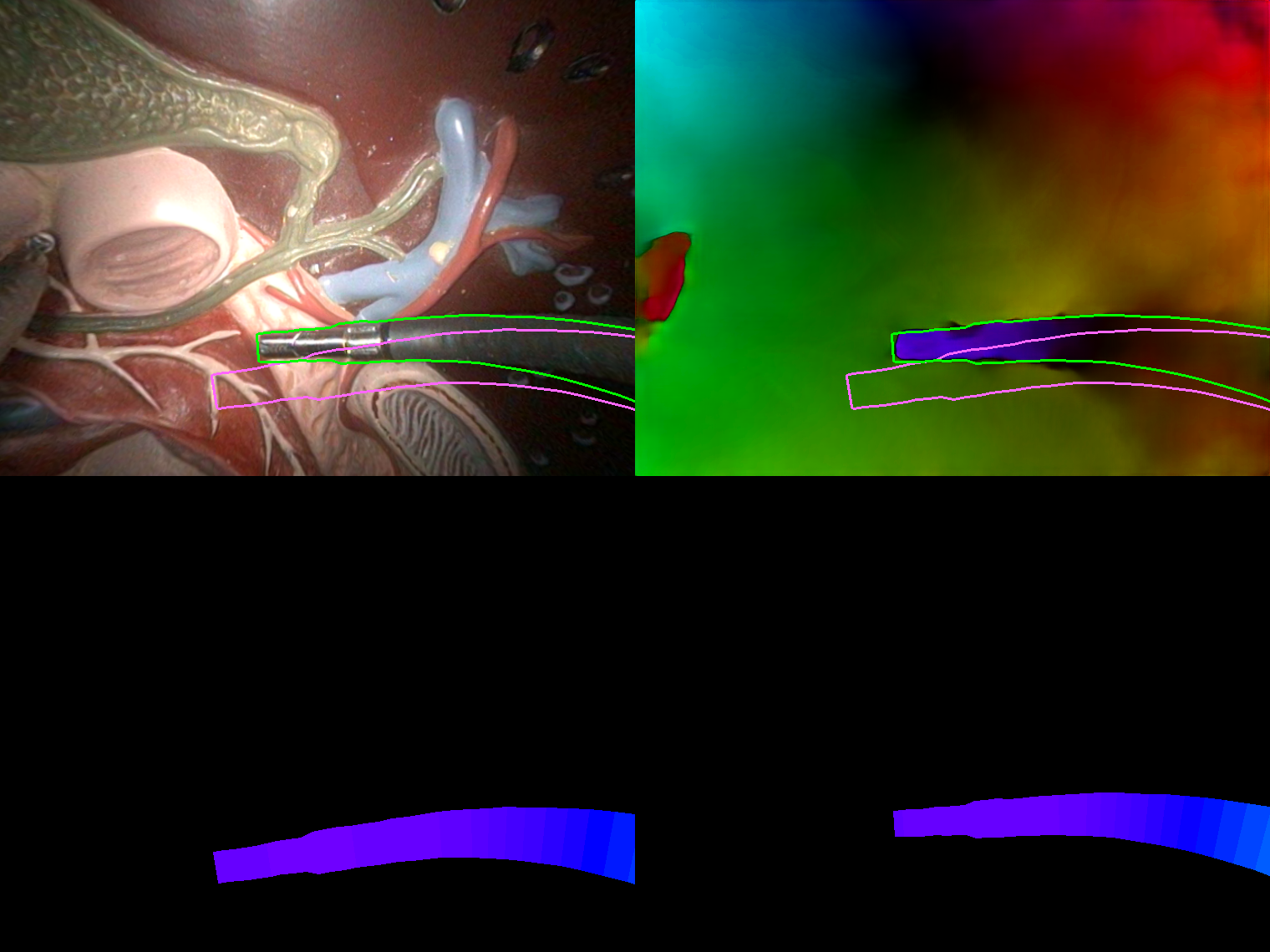

Supplement: Supplementary file 2 [file Data_Sheet_1.ZIP › 22_complete.tiff]

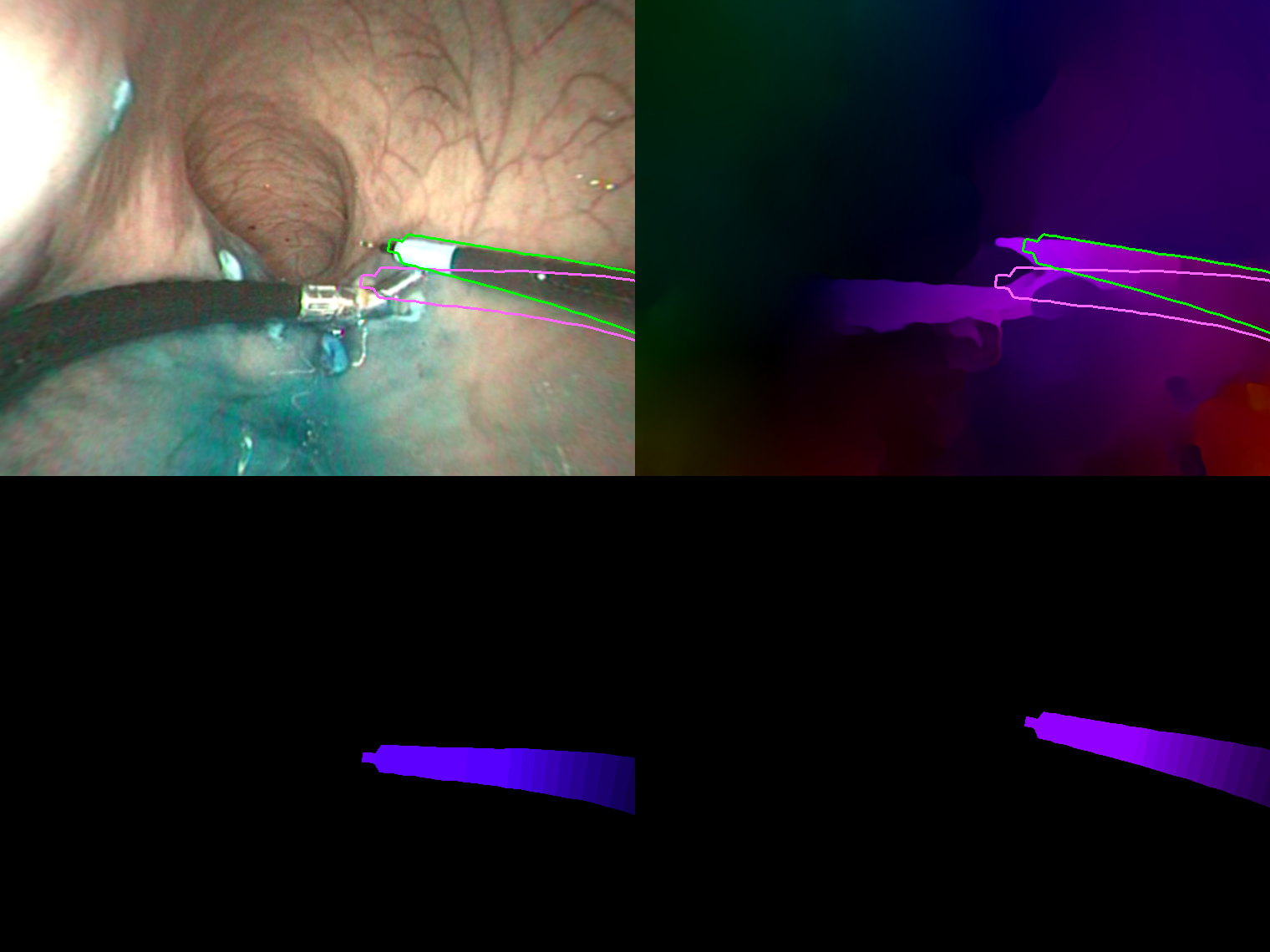

Supplement: Supplementary file 2 [file Data_Sheet_1.ZIP › 23_complete.tiff]

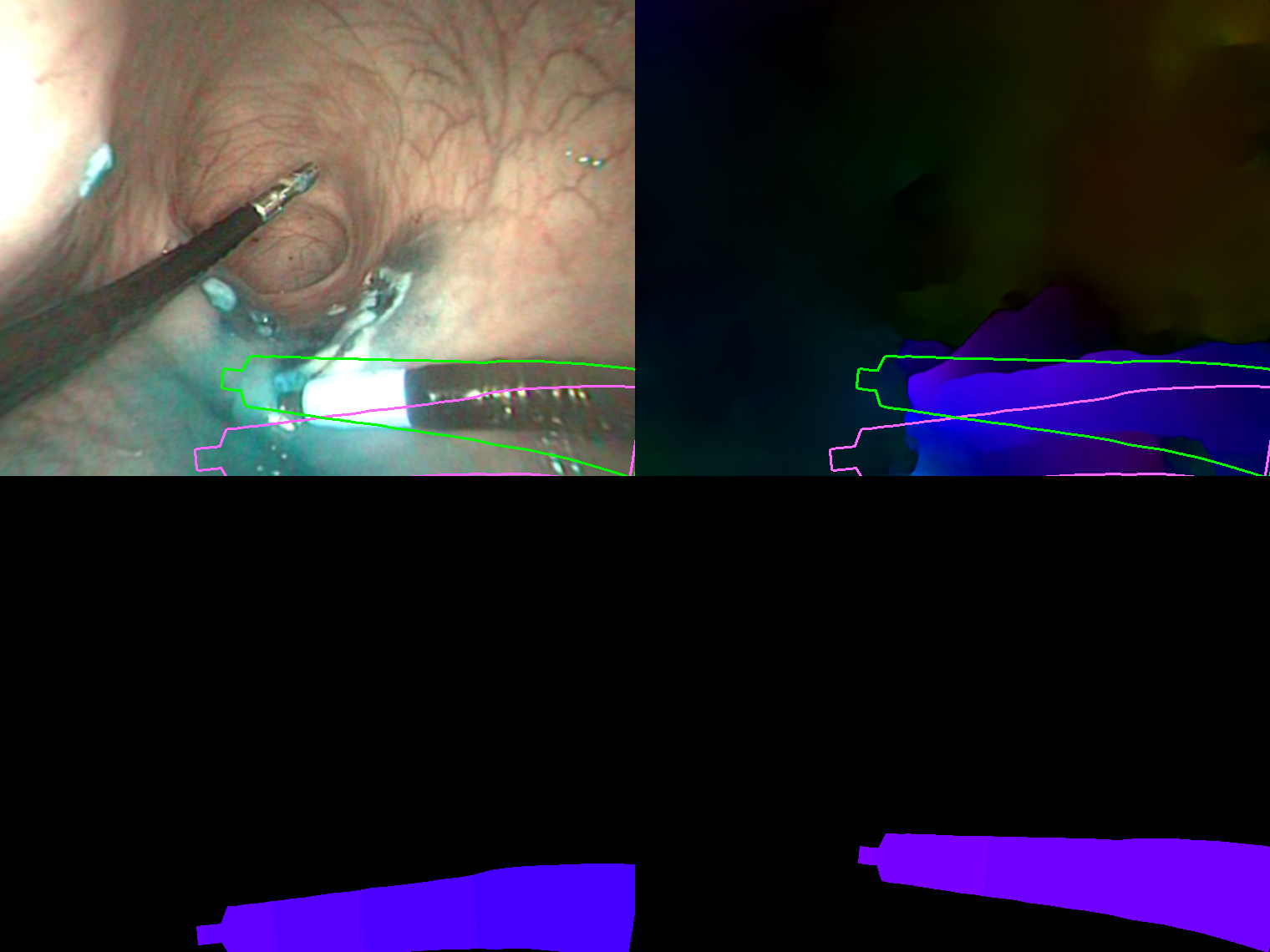

Supplement: Supplementary file 2 [file Data_Sheet_1.ZIP › 24_complete.tiff]

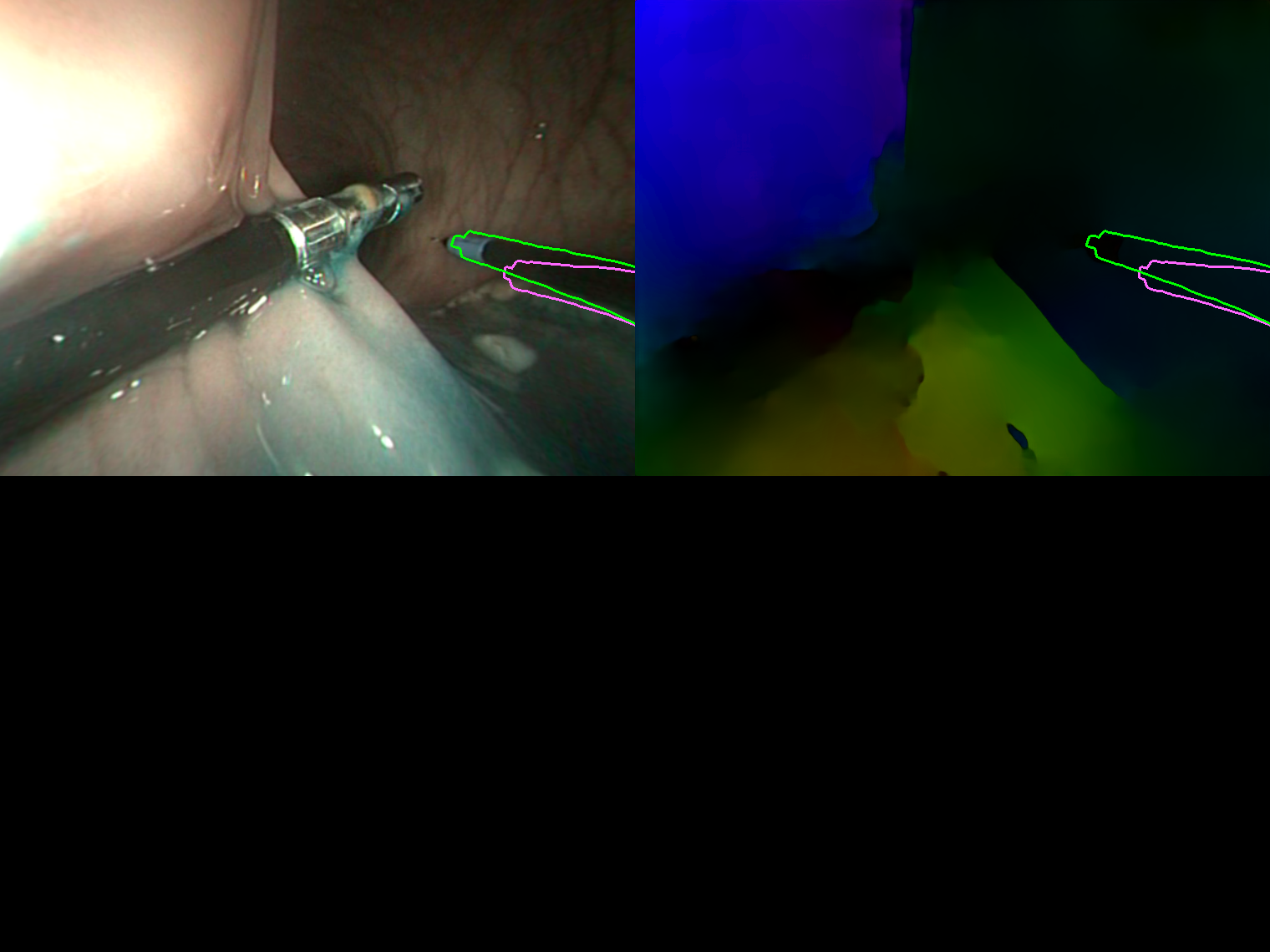

Supplement: Supplementary file 2 [file Data_Sheet_1.ZIP › 25_complete.tiff]

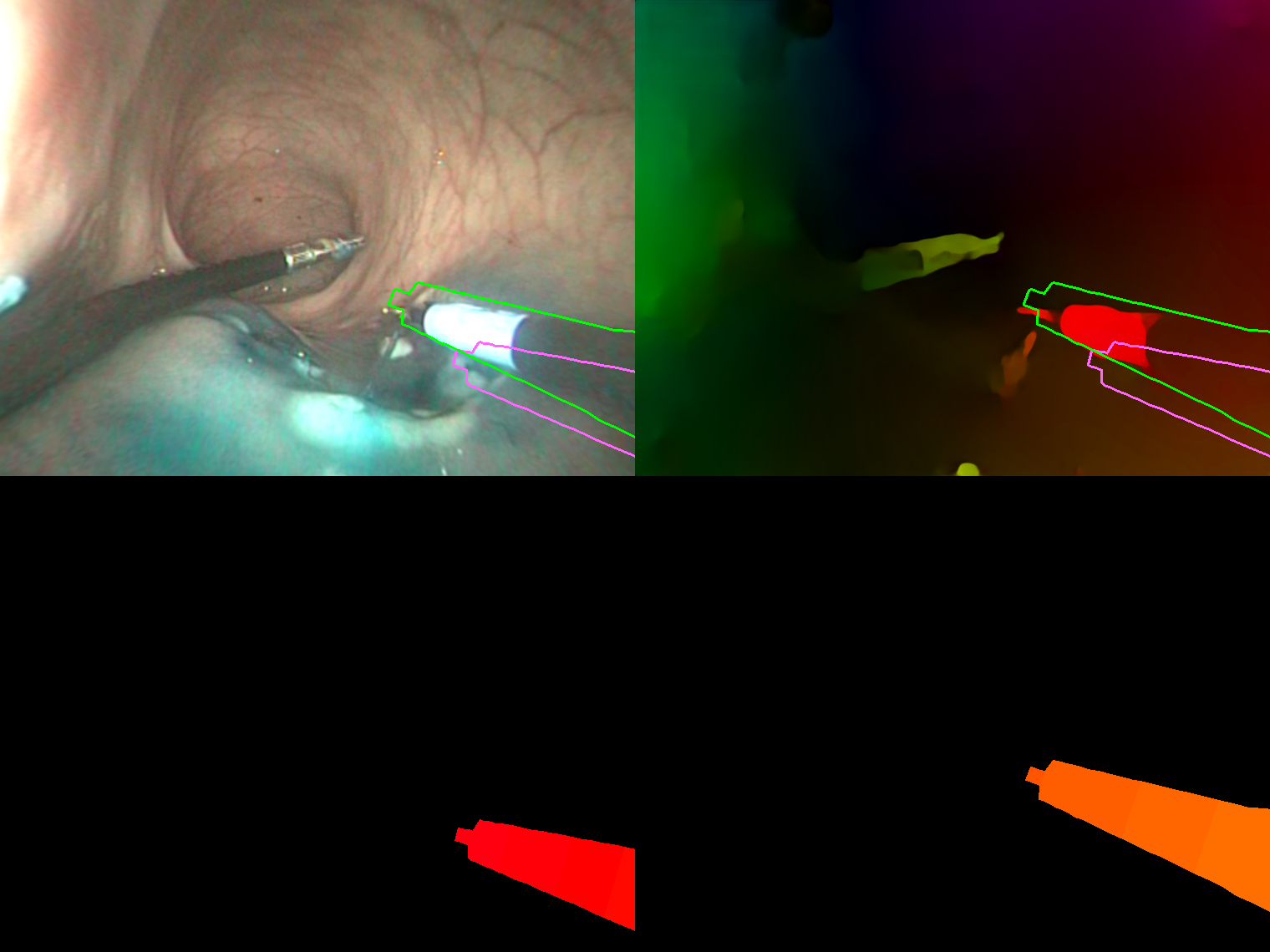

Supplement: Supplementary file 2 [file Data_Sheet_1.ZIP › 26_complete.tiff]

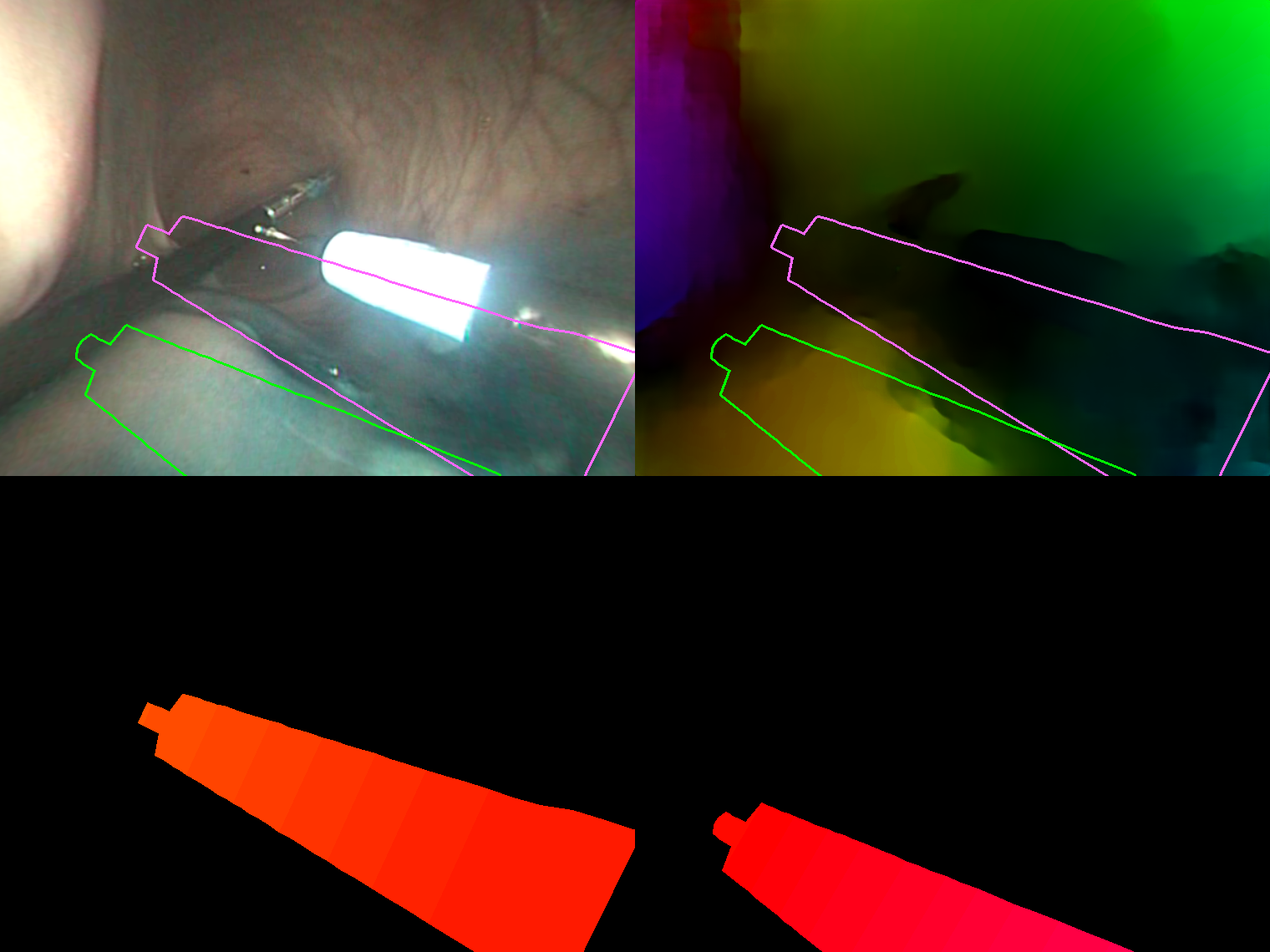

Supplement: Supplementary file 2 [file Data_Sheet_1.ZIP › 27_complete.tiff]

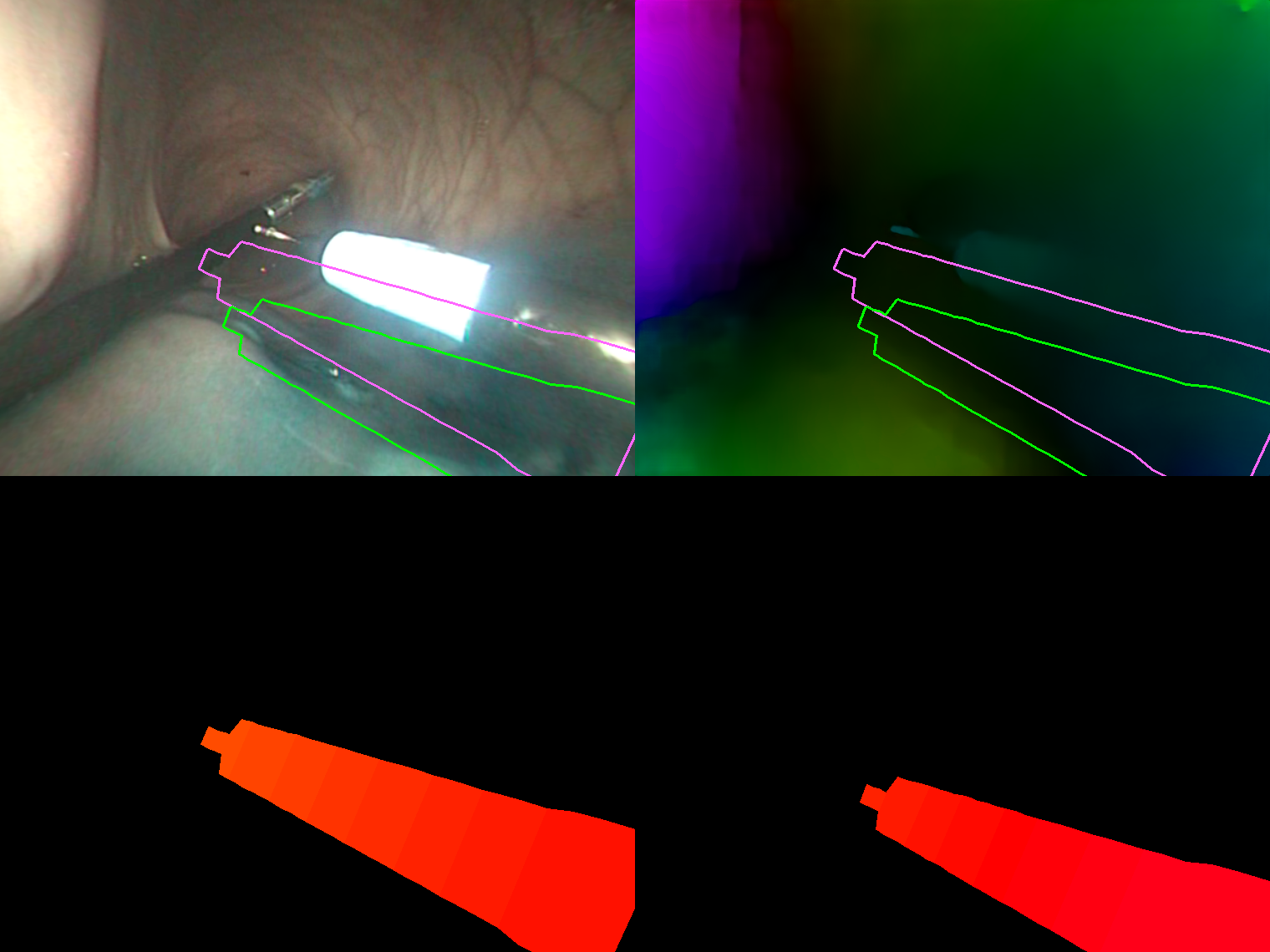

Supplement: Supplementary file 2 [file Data_Sheet_1.ZIP › 28_complete.tiff]

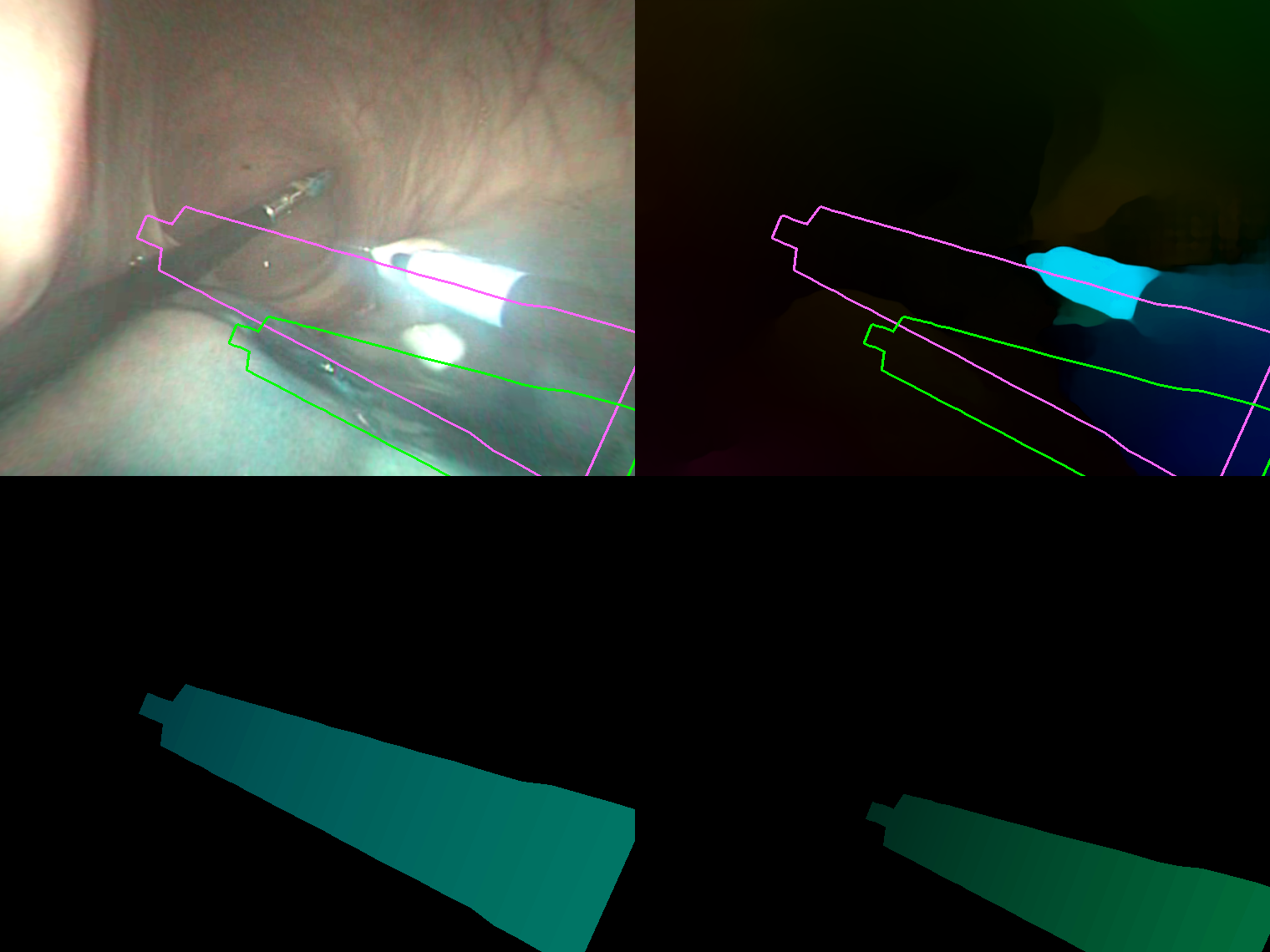

Supplement: Supplementary file 2 [file Data_Sheet_1.ZIP › 29_complete.tiff]

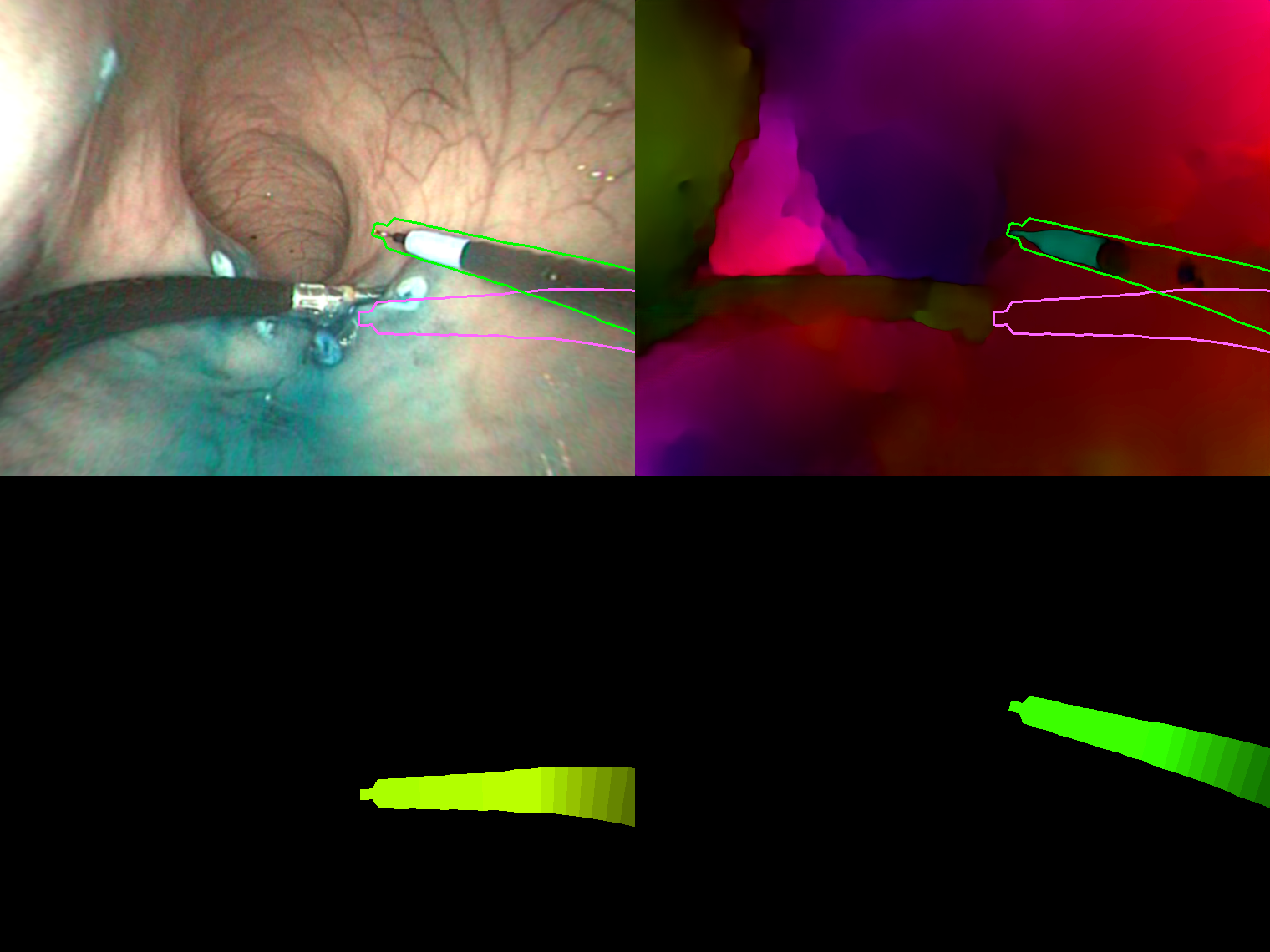

Supplement: Supplementary file 2 [file Data_Sheet_1.ZIP › 2_complete.tiff]

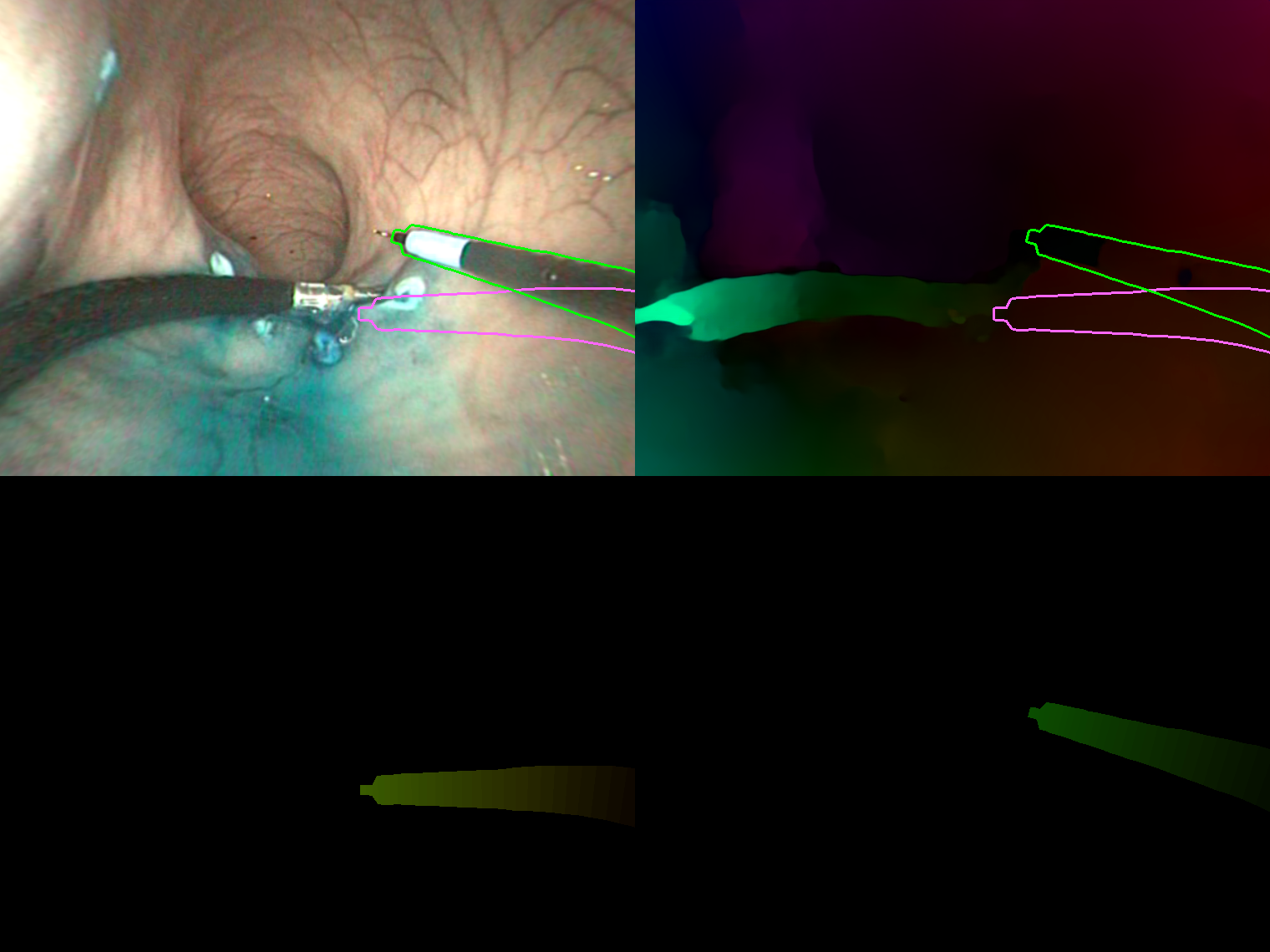

Supplement: Supplementary file 2 [file Data_Sheet_1.ZIP › 3_complete.tiff]

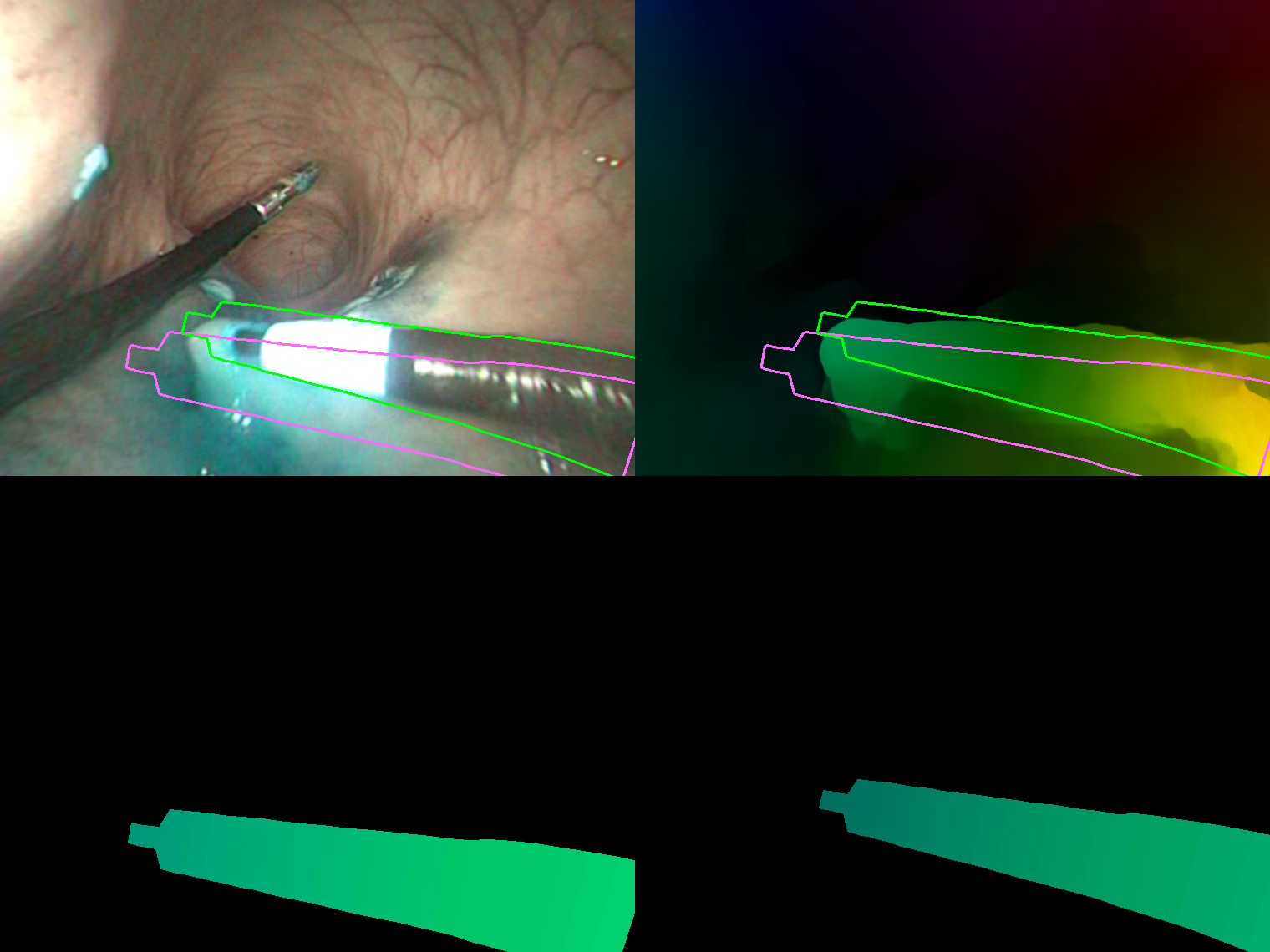

Supplement: Supplementary file 2 [file Data_Sheet_1.ZIP › 4_complete.tiff]

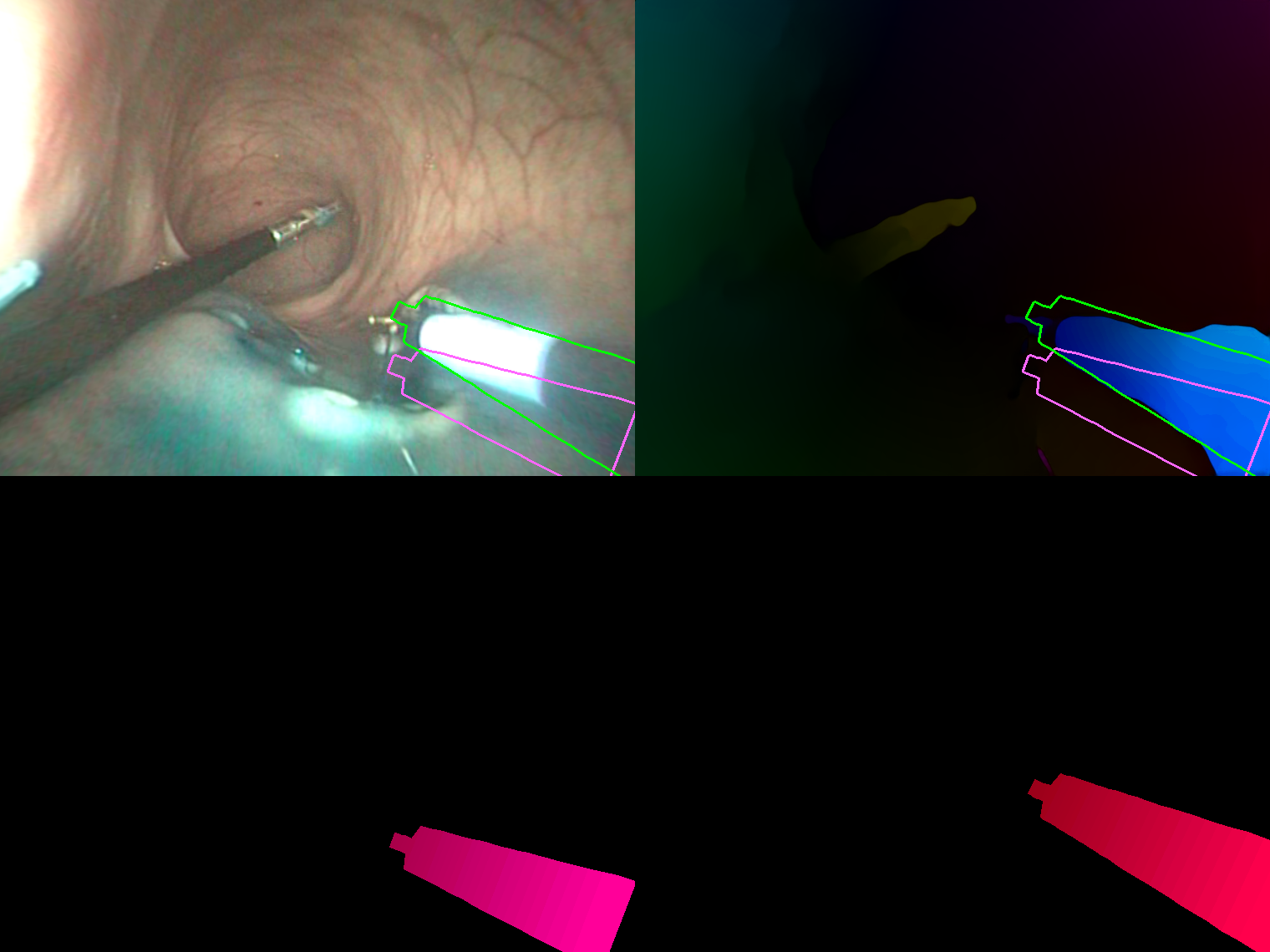

Supplement: Supplementary file 2 [file Data_Sheet_1.ZIP › 5_complete.tiff]

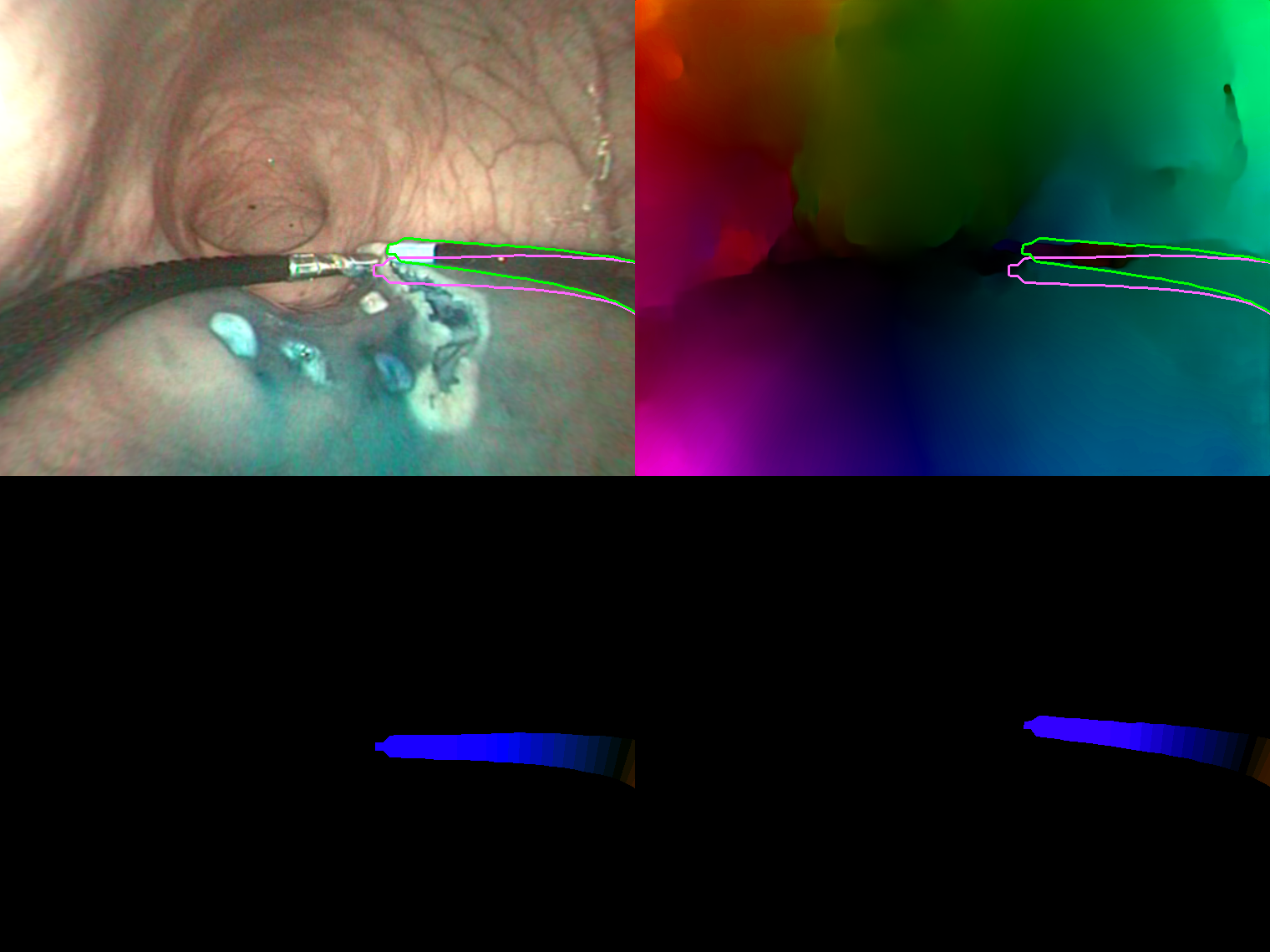

Supplement: Supplementary file 2 [file Data_Sheet_1.ZIP › 6_complete.tiff]

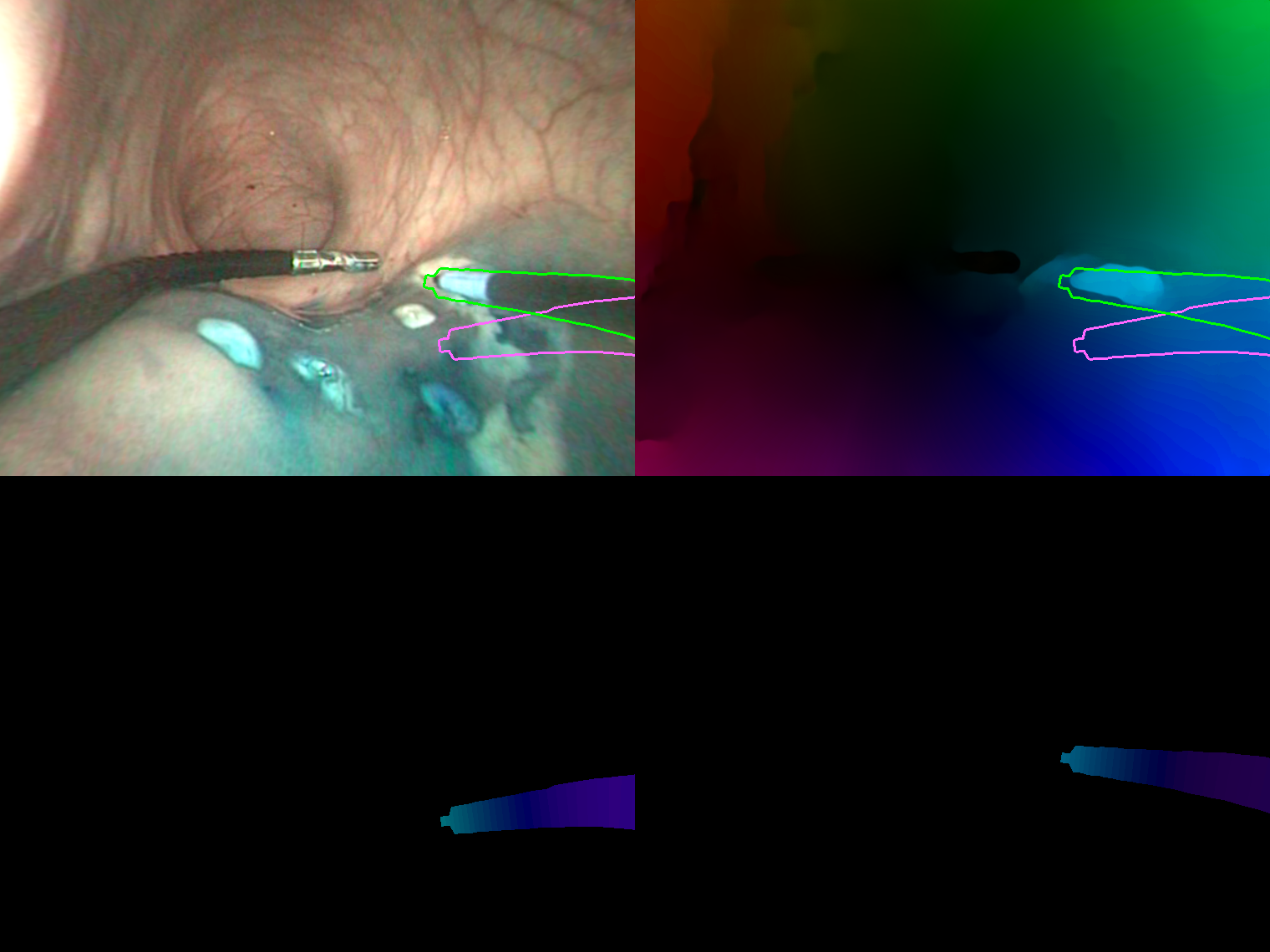

Supplement: Supplementary file 2 [file Data_Sheet_1.ZIP › 7_complete.tiff]

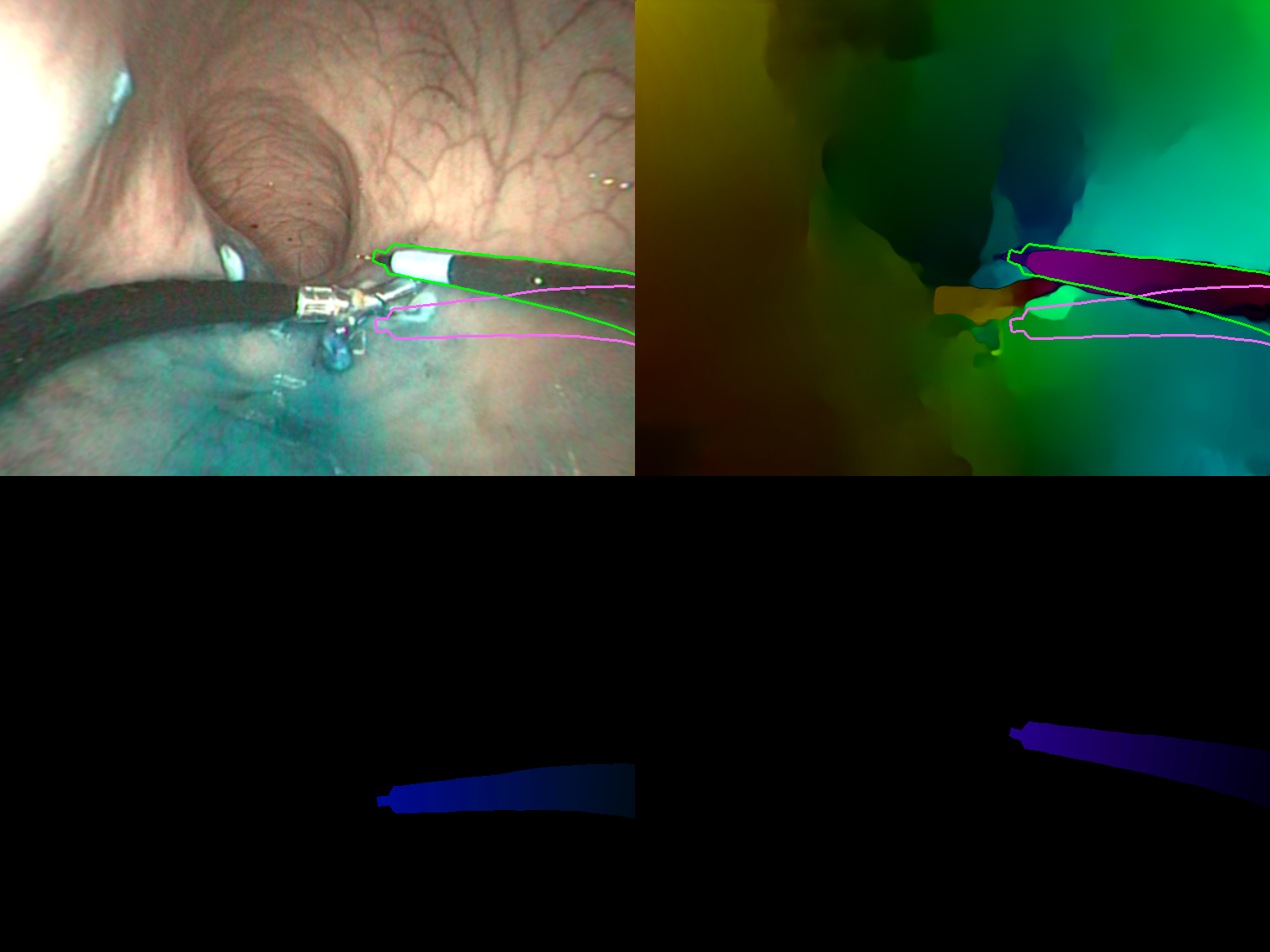

Supplement: Supplementary file 2 [file Data_Sheet_1.ZIP › 8_complete.tiff]

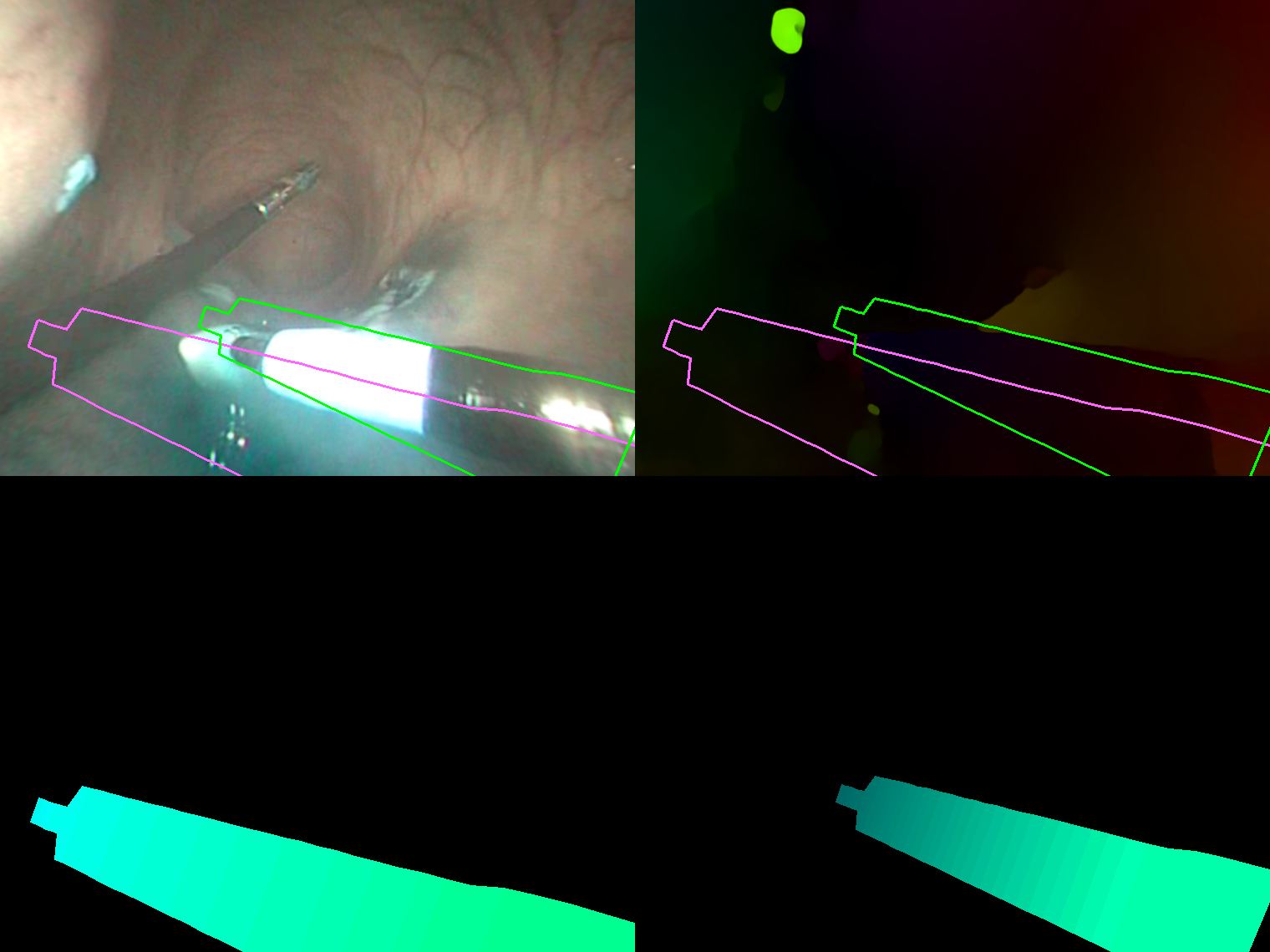

Supplement: Supplementary file 2 [file Data_Sheet_1.ZIP › 9_complete.tiff]
